# Hsa-microRNA-27b-3p inhibits hepatocellular carcinoma progression by inactivating transforming growth factor-activated kinase-binding protein 3/nuclear factor kappa B signalling

**DOI:** 10.1186/s11658-022-00370-4

**Published:** 2022-09-23

**Authors:** Jingyuan Wen, Zhao Huang, Yi Wei, Lin Xue, Yufei Wang, Jingyu Liao, Junnan Liang, Xiaoping Chen, Liang Chu, Bixiang Zhang

**Affiliations:** 1 Hepatic Surgery Center and Hubei Key Laboratory of Hepato-Biliary-Pancreatic Diseases, Tongji Hospital, Tongji Medical College, Huazhong University of Science and Technology, 1095 Jiefang Avenue, Wuhan, 430030 China; 2 Clinical Medical Research Center of Hepatic Surgery at Hubei Province, Wuhan, China; 3 Key Laboratory of Organ Transplantation, Ministry of Education; Key Laboratory of Organ Transplantation, National Health Commission; Key Laboratory of Organ Transplantation, Chinese Academy of Medical Science, Wuhan, China

**Keywords:** HCC, *Hsa-miR-27b*, *NF-кB*, *TAB3*, Oncolytic adenovirus

## Abstract

**Background:**

MicroRNAs (miRNAs) play crucial roles in the development of hepatocellular carcinoma (HCC). Hsa-microRNA-27b-3p (*hsa-miR-27b*) is involved in the formation and progression of various cancers, but its role and clinical value in HCC remain unclear.

**Methods:**

The expression of *hsa-miR-27b* in HCC was examined by quantitative real-time PCR (qRT-PCR) and in situ hybridization (ISH) assays of clinical samples. Cell Counting Kit-8 assays (CCK-8), 5-ethynyl-2′-deoxyuridine (EdU) incorporation assays, Transwell assays, filamentous actin (*F-actin*) staining and western blot analyses were used to determine the effects of *hsa-miR-27b* on HCC cells in vitro. Subcutaneous xenograft and lung metastatic animal experiments were conducted to verify the role of *hsa-miR-27b* in HCC in vivo. In silico prediction, qRT-PCR, western blot, anti-Argonaute 2 (*AGO2*) RNA immunoprecipitation (RIP) and dual luciferase reporter assays were applied to identify the target genes of *hsa-miR-27b*. To detect the impacts of *hsa-miR-27b* on nuclear factor kappa B (*NF-кB*) signalling cascades mediated by transforming growth factor-activated kinase-binding protein 3 (*TAB3*), we performed qRT-PCR, western blot assays, immunofluorescence staining, immunohistochemistry (IHC) and dual-luciferase reporter assays. Recombinant oncolytic adenovirus (Onco^Ad^) overexpressing *hsa-miR-27b* was constructed to detect their therapeutic value in HCC.

**Results:**

The expression of *hsa-miR-27b* was lower in HCC than in adjacent non-tumourous tissues (ANTs), and the reduced expression of *hsa-miR-27b* was associated with worse outcomes in patients with HCC. *Hsa-miR-27b* significantly inhibited the proliferation, migration, invasion, subcutaneous tumour growth and lung metastasis of HCC cells. The suppression of *hsa-miR-27b* promoted the nuclear translocation of *NF-κB* by upregulating *TAB3* expression. *TAB3* was highly expressed in HCC compared with ANTs and was negatively correlated with the expression of *hsa-miR-27b*. The impaired cell proliferation, migration and invasion by *hsa-miR-27b* overexpression were recovered by ectopic expression of *TAB3*. Recombinant Onco^Ad^ with overexpression of *hsa-miR-27b* induced anti-tumour activity compared with that induced by negative control (NC) Onco^Ad^ in vivo and in vitro.

**Conclusions:**

By targeting *TAB3*, *hsa-miR-27b* acted as a tumour suppressor by inactivating the *NF-кB* pathway in HCC in vitro and in vivo, indicating its therapeutic value against HCC.

**Graphical Abstract:**

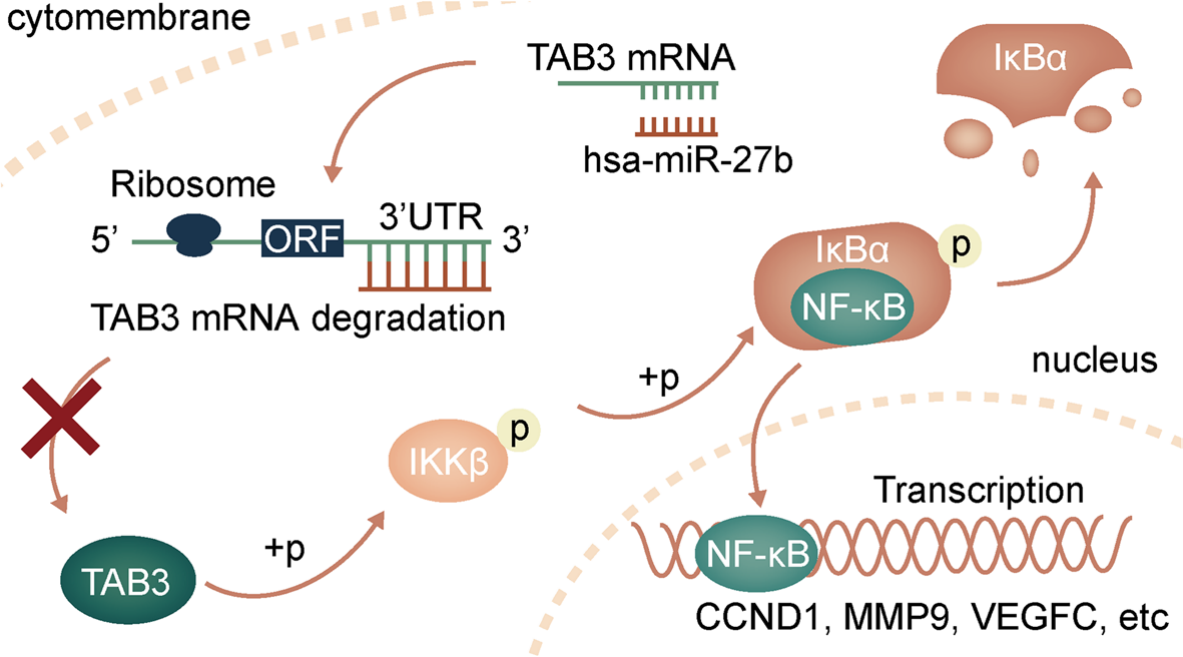

**Supplementary Information:**

The online version contains supplementary material available at 10.1186/s11658-022-00370-4.

## Background

Hepatocellular carcinoma (HCC) is one of the most malignant tumours with a poor prognosis, and the incidence of HCC is still increasing globally [1]. It is critical to elucidate the underlying molecular mechanism of HCC formation and progression, to identify new potential diagnostic biomarkers and to develop effective therapeutic interventions for HCC [2]. miRNAs, small non-coding RNAs that consist of approximately 22 nucleotides, were found to have crucial roles in carcinogenesis [3]. miRNAs can regulate multiple genes by targeting messenger RNA (mRNA) transcripts, leading to mRNA translational repression or degradation [4]. The expression patterns of miRNAs are tissue specific; thus, miRNAs can provide a basis for an in-depth understanding of the mechanisms of tumour progression [5, 6].

Many studies have demonstrated that *hsa-miR-27b* acts as an oncogene or a tumour suppressor in different tumour types [7–9]. Studies have shown that the high expression of *hsa-miR-27b* promotes the proliferation and invasion of cervical cancer and breast cancer [10, 11]. However, *hsa-miR-27b* could increase the anti-tumour immune response by regulating proliferator-activated receptor γ expression in neuroblastoma cells [12]. In addition, *hsa-miR-27b* inhibited gastric cancer cell proliferation, migration and invasion in the liver [13]. With respect to HCC, *hsa-miR-27b* has been reported to be overexpressed in HCC and mediate the proliferation, cell-cycle progression and apoptosis of HCC cells [14]. He et al. showed that *hsa-miR-27b* was frequently upregulated in HCC and promoted the migration and invasion of cancer cells [15]. However, some studies showed the opposite results. Liang et al. explored the clinical importance of *hsa-miR-27b* in HCC and found that the expression of *hsa-miR-27b* in tumour tissues was lower than that in ANTs [16]. *Hsa-miR-27b*, which was found to be deleted in liver cancer, sensitized HCC cells to chemotherapy by activating *p53*-dependent apoptosis and reducing cytochrome P450 family 1 subfamily B member 1-mediated drug detoxification [17]. Interestingly, Fu et al. showed that premature *hsa-miR-27b* was frequently upregulated, while mature *hsa-miR-27b* was downregulated in HCC tissues compared with ANTs. Mechanistically, long non-coding RNA DNAJC3 anti-sense RNA 1 sponged premature *hsa-miR-27b* in the nucleus to suppress its maturation and promote cell proliferation [18]. To date, the expression pattern of *hsa-miR-27b* and its exact roles in HCC are unclear, and therapeutic applications of *hsa-miR-27b* in HCC remain to be elucidated.

The *NF-кB* pathway plays a critical role in regulating cellular processes such as immune responses [19], inflammatory reactions (20), apoptosis [21], proliferation and differentiation [22]. Dysfunction of this pathway is associated with the progression of numerous cancers and immune disorders [23]. Under normal conditions, *NF-кB* is inactivated by binding with the inhibitors of NF-кB (*IκB*) and is sequestered in the cytoplasm [24]. Upon certain cell stimulation, such as tumour necrosis factor alpha (*TNF-α*), interleukin-1 and lipopolysaccharide, the activated IκB kinase (*IKK*) complex induces the phosphorylation and degradation of *IκB* proteins, which subsequently liberates *NF-κB* to the nucleus and initiates transcriptional processes [19, 25]. *TAB3* is dysregulated in a variety of cancers [26–28] and participates in the activation of the *NF-кB* pathway [29, 30]. Alfredo Criollo et al. reported that overexpression of *TAB3* suppressed, while depletion of *TAB3* triggered, autophagy in HeLa cells [31]. Overexpression of *TAB3* induced constitutive cellular activation of *NF-κB* signalling [29]. Chen et al. demonstrated that *TAB3* was upregulated in ovarian cancer cells and tissues and predicted poor prognosis of patients [32]. In addition, knockdown of *TAB3* could retain RELA proto-oncogene (*p65*) in the cytoplasm, indicating inactivation of the *NF-κB* pathway [32]. Mechanistically, *TAB3* phosphorylates the *IKK* complex by forming the transforming growth factor-activated kinase 1 (*TAK1*)/transforming growth factor-activated kinase-binding protein 2 (*TAB2*)/*TAB3* complex, which leads to the degradation of *IкB* and the nuclear translocation of *NF-кB* [33]. Although the functions of *TAB3* in *NF-кB* signalling have been well studied, the underlying role of *TAB3* in the progression of HCC remains unclear.

In this research, the decreased expression of *hsa-miR-27b* in HCC tissues was validated by analysis of patient data from our own clinical samples and public database. We demonstrated that *hsa-miR-27b* exerted a tumour-suppressor effect in HCC by targeting *TAB3*. Moreover, an oncolytic virus carrying *hsa-miR-27b* succeeded in suppressing tumour growth in a xenograft animal model, indicating the therapeutic role of *hsa-miR-27b* in patients with HCC.

## Materials and methods

### Patients and tissue specimens

A total of 71 pairs of HCC and ANTs from January 2015 to December 2016 were collected from patients with HCC who underwent hepatectomy at the Hepatic Surgery Center, Tongji Hospital of Huazhong University of Science and Technology (HUST) (Wuhan, China). HCC diagnosis was confirmed by histopathology. The median follow-up period was 27 months (range, 1–45 months). Forty-three pairs of snap-frozen HCC specimens were used to obtain total RNA and protein. All procedures were approved by the Ethics Committee of Tongji Hospital, HUST, and conducted according to the principles of the Declaration of Helsinki. Prior written and informed consent was obtained from each patient.

### Cell lines and culture

The HCC cell lines HLF, MHCC97H and HCCLM3 were obtained from the Liver Cancer Institute, Zhongshan Hospital, Fudan University, as previously described [34]. The human normal liver cell line WRL68 (PWE-HU129) was purchased from Meilune Biological Technology Co. (Dalian, China). The HCC cell lines Hep3B (GDC0070) and Huh7 (GDC0134) were purchased from the China Center for Type Culture Collection (CCTCC, Wuhan, China). The HCC cell line PLC/PRF/5 (TCHu119) was purchased from the Cell Bank of the Chinese Academy of Sciences (Shanghai, China). For all cell lines, short tandem repeat (STR) analysis was performed to confirm cell identity. All cell lines were maintained in Dulbecco’s modified Eagle medium (DMEM) (HyClone, UT, USA) with 10% foetal bovine serum (FBS) (Gibco) at 37 °C in a 5% CO_2_ humid atmosphere.

### ISH and IHC analysis

ISH was performed using the ISH Kit (Boster, Bioengineering Company, Wuhan, China). All procedures were performed following the manufacturer’s instructions. Samples were stained with haematoxylin, dehydrated with alcohol, washed with xylene and sealed with flavour sealing tablets. Oligo (5′ digoxin-ACAAAGTTCTGTAGTGC-ACTGA) was used as an ISH probe for* hsa-miR-27b*. IHC was performed as described previously [35]. The dilutions of antibodies for the IHC procedure are listed in Additional file 1: Table S1. Representative images of ISH and IHC were captured and processed using a DM2300 microscope and ScopeImage 9.0 software (Nanjing Jiangnan Novel Optics Co., Ltd., China). ISH and IHC staining scores were independently determined by three pathologists without prior knowledge of patient information. The overall scores were defined by multiplying the percentage of positive cells by the staining intensity score as described previously [35].

### RNA isolation and qRT-PCR

Total RNA from human specimens and cancer cells was extracted by TRIzol reagent (Invitrogen, Carlsbad, CA, USA) according to the manufacturer’s protocol. We used a reverse-transcription system (Vazyme, Nanjing, China) and miRcute Plus miRNA First-Strand cDNA Synthesis (Tiangen, Beijing, China) kits to generate cDNA of mRNA and miRNA, respectively. Relative quantification of *hsa-miR-27b* and the mRNA expression levels were determined by a miRcute Plus miRNA qPCR Detection Kit (Tiangen, Beijing, China) and SYBR Green PCR kit (Vazyme, Nanjing, China) according to the manufacturers’ protocol. *GAPDH* and *U6* small nuclear RNA were used as internal controls for mRNA and miRNA, respectively. We used the comparative *C*_T_ (2^−∆∆CT^) method to perform relative quantification analysis. The primers used in this study are listed in Additional file 1: Table S2.

### Cell transfection and infection

HCC cells were seeded in six-well plates at 70% density and cultured overnight. *Hsa-miR-27b* mimics and inhibitors and their respective NC (RiboBio, Guangzhou, China) were transfected with Lipofectamine 3000 (Invitrogen, USA). Small interference RNA (siRNA) targeting *TAK1* was used to transiently knock down its expression in HCC cells. si-NC: 5′-UUGUACUACACAAAAGUACUG-3′; si1-*TAK1*: 5′-GGUAGUAAUUACAGUGAAA-3′; si2-*TAK1*: 5′-CCCGTGTGAACCATCCTAATA-3′.

Lentiviruses for stably overexpressing or knocking down *TAB3* were generated by Shanghai GeneChem Co., Ltd. (Shanghai, China) and used to infect the indicated cells for 48 h. Transfected cells were then selected by culturing in medium containing 2.5 μg/ml puromycin for 2 weeks. The small hairpin RNA (shRNA) target sequences for knocking down the indicated genes were as follows: scramble: 5′-GCCTAAGGTTAAGTCGCCCTCG-3′; sh-*TAB3*: 5′-CCTCCTTCATACATGCACATA-3′.

### CCK-8 assays

Cells were seeded into 96-well plates at 1500 cells per well and incubated at 37 °C. The culture medium was changed to 100 μl of 10% CCK-8 (Dojindo, Kumamoto, Japan) solution at the indicated time and incubated in a cell incubator for 2 h. Optical density (OD) was measured by a Universal Microplate Reader ELx 800 (Bio-Tek, USA) at a wavelength of 450 nm. For each group, the absorbance values were determined by five replicates. After extraction of the blank value, the average gross OD values were used for data analysis.

### Transwell assays

Transwell chambers (8 μm pore size, Corning, NY, USA) were used for cell migration assays. In Transwell migration assays, HCC cells suspended in 200 μl of serum-free DMEM (HyClone, UT, USA) were added to the upper chambers, and the lower chambers were filled with 500 μl of DMEM with 10% FBS. In the Transwell invasion assay, before cells were seeded in the upper chamber, chambers were coated with Matrigel (BD Biosciences, NJ, USA) for 3 h. Following 24 h (migration assays) or 48 h (invasion assays) of incubation at 37 °C, the migrated or invaded cells were fixed with 4% paraformaldehyde and stained with 1% crystal violet solution. Stained cells were counted in three random microscopic fields per well by a Nikon Digital ECLIPSE C1 system (Nikon Corporation).

### *F-actin* assay

The indicated cells were seeded in 96-well plates at a density of 1000 cells per well and cultured overnight. Cells were then fixed with 4% paraformaldehyde for 15 min at room temperature. After two washes with phosphate-buffered saline (PBS), 100 µl of Alexa Fluor 555-conjugated phalloidin (Life Technologies, USA) was added into each well for 1 h at room temperature. PBS washes were performed twice, and the nuclei were counterstained with DAPI. The cells were then observed using the EVOS FL auto-imaging system (Life Technologies, USA).

### Dual-luciferase reporter assay

The DNA sequence containing the predicted binding site with *hsa-miR-27b* in the 3′-untranslated region (3′UTR) of *TAB3* or the 3′UTR-*TAB3* mutant was cloned into the psiCHECK-2-vector (Promega, Madison, WI, USA). Approximately 1 × 10^5^ cells per well were seeded in 24-well plates. After 24 h, the recombinant plasmid psiCHECK-2-3′UTR-*TAB3* or psiCHECK-2-3′UTR-*TAB3* mutant was co-transfected into cells with *hsa-miR-27b* mimics, *hsa-miR-27b* inhibitors or their respective NC using Lipofectamine 3000 (Invitrogen, USA). Total cell protein was extracted with Passive Lysis Buffer (Promega, Madison, WI, USA), and luciferase activity was determined using the Dual-Luciferase Reporter 1000 Assay System (Promega, Madison, WI, USA) with a GloMax 20/20 luminometer (Promega, Madison, WI, USA). Renilla luciferase values were normalized against firefly luciferase activity, and the ratio of Renilla-to-firefly luciferase activity is presented. For *NF-кB* signalling activation analysis, *NF-кB* luciferase reporter plasmid containing the minimal promoter with multiple tandem *NF-кB* binding sites (pNF-кB-Luc, Clontech, Palo Alto, CA, USA) and its control vector (pTAL-Luc, Clontech) were transfected into HCC cells by Lipofectamine 3000 (Invitrogen, USA). The indicated cell lines were treated with *TNF-α* (10 ng/ml) (MCE, USA).

### Immunofluorescence staining

HCC cells were cultured on sterile coverslips in 24-well plates and treated with 20 ng/ml *TNF-α* (MCE, USA) for 10 min. After treatment, the cells were fixed in 4% paraformaldehyde for 15 min at room temperature, permeabilized with 0.5% Triton X-100 solution for 10 min and incubated with primary antibody at 4 °C overnight. Alexa Flour 488-conjugated anti-rabbit IgG and Alexa Flour 555-conjugated anti-mouse IgG (Beyotime Institute of Biotechnology, Shanghai, China) were used to incubate cells for 1 h at 37 °C. Nuclei were stained with DAPI (Wuhan Goodbio Biotechnology Co., Ltd., Wuhan, China). Images were captured by an EVOS FL auto imaging system (Life Technologies, USA).

### Animal experiments

All BALB/c nude mice were purchased from Beijing HFK Bioscience Co., Ltd., and maintained under specific-pathogen-free conditions. For the xenograft tumour model, 1 × 10^6^ indicated tumour cells were suspended in 100 μl of serum-free DMEM and inoculated subcutaneously into the flanks of 4-week-old nude mice. All experimental mice were monitored for 30 days and sacrificed to compare the volume and weight of subcutaneous tumours. For the in vivo metastasis assays, 1 × 10^6^ cells in 100 μl of serum-free DMEM were injected into the tail vein of nude mice. After 2 months, the nude mice were sacrificed, and the lungs were dissected. All of the metastatic foci in the lung were calculated to evaluate the development of pulmonary metastasis.

For the Onco^Ad^-mediated treatment of liver cancer, Hep3B tumour xenografts were established by subcutaneous inoculation of 5 × 10^5^ cells into the right flank of 4-week-old nude mice. Twenty-one days after inoculation, the animals were randomly divided into three groups (*n* = 7 mice per group) and intra-tumourally injected with either PBS solution, Onco^Ad^ NC or Onco^Ad^ *hsa-miR-27*b (diluted in PBS) at multiplicities of infection (MOIs) of 5 × 10^8^. The injections were performed once every other day for a total of four injections. Tumours were measured every 3 days, and volume was calculated by the following formula: volume = (length × width^2^)/2.

### Recombinant adenovirus production

Onco^Ad^ *hsa-miR-27b* and Onco^Ad^ NC were constructed by Shanghai GeneChem Co., Ltd. (Shanghai, China). The *u6-hsa-miR-27b* cassette was cloned into the oncolytic adenoviral vector with the E1B 55-KD gene deletion. The recombinant adenovirus was transfected into 293T cells for amplification. The viral particles were collected, and the titre was detected according to the manufacturer’s operating instructions after purification.

### Cytotoxicity assay

Hep3B and WRL68 cells were used for cytotoxicity assays in 96-well or 24-well plates by CCK-8 assays or crystal violet staining. Briefly, cells were pre-seeded in 96-well plates and cultured overnight. Onco^Ad^ at the indicated MOIs was used to treat cells for 24 h. Cell viability was measured by CCK-8 assays. Additionally, cells were prepared in the same manner in 24-well plates and cultured for 48 h. Then, the cells were fixed with 4% paraformaldehyde and stained with crystal violet staining solution.

### Nuclear and cytoplasmic extraction assay

A total of 1 × 10^6^ HCC cells were collected in a 1.5 ml microcentrifuge tube and then centrifuged at 500*g* for 5 min. The cells were washed by suspending the cell pellet with PBS. The supernatant was removed, leaving the cell pellet as dry as possible. Cytoplasmic and nuclear fraction protein extraction was performed using an NE-PER nuclear and cytoplasmic extraction reagent kit (Thermo Fisher Scientific, USA) according to the instructions.

### In silico analysis

The miRNA expression profiles were retrieved from The Cancer Genome Atlas (TCGA) database (https://tcga-data.nci.nih.gov/tcga/) and the Gene Expression Omnibus (GEO) database (http://www.ncbi.nlm.nih.gov/gds). Normalization and batch effect elimination were carried out for the two gene datasets using the Limma package in R. Statistical significance was calculated using the Kruskal–Wallis test. The correlation between the survival time and *TAB3* expression in patients with HCC was assessed using the GEPIA website (http://gepia.cancer-pku.cn/). The miRDB (http://mirdb.org/miRDB), miRWalk (http://www.umm.uniheidelberg.de/apps-/zmf/mirwalk/) and TargetScan (http://www.targetscan.org/vert_71/) databases were used for miRNA target‐gene prediction.

### EdU incorporation assays

HCC cells were seeded in 96-well plates at a density of 1000 cells per well and incubated overnight. Then, the cells were transfected with the indicated mimics or inhibitors for 72 h and subjected to an EdU incorporation assay using a Cell-Light EdU Apollo 567 In Vitro Imaging Kit (RiboBio, Guangzhou, China) according to the manufacturer’s instructions.

### RIP assay

We performed RIP using the Magna RIP RNA-Binding Protein Immunoprecipitation Kit (Millipore, Darmstadt, Germany) following the manufacturer’s instructions. A detailed description was published previously [35].

### Co-immunoprecipitation (co-IP)

The experiments were performed as described previously [36]. Briefly, a total of 1 × 10^7^ cells were lysed with IP lysis buffer for 20 min at 4 °C (IP-lysis buffer: 50 mM Tris–HCl, 150 mM NaCl, 1% Triton X-100, 1 mM EDTA, 10% glycerol and protease inhibitor cocktail, pH 7.4) and then centrifuged at 13,000 rpm for 10 min. The supernatant was incubated with 30 μl of pre-cleared protein G-conjugated agarose (GE Healthcare Life Sciences) for 4 h at 4 °C. The indicated antibodies were added into cleared supernatants overnight at 4 °C. One per cent of the lysate fraction was reserved as an input. The next day, 25 μl of protein G-conjugated agarose was used to precipitate the antibody–protein mixture at 4 °C for 4 h. The beads were then washed five times in IP wash buffer (50 mM Tris–HCl, 300 mM NaCl, 1% Triton X-100 and 1 mM EDTA, pH 7.4) to remove unbound antibodies. The eluate was separated from the beads by heating at 95 °C for 10 min.

### Statistical analyses

We used SPSS 19.0 (IBM, Chicago, IL, USA) or Prism 6.0 (GraphPad Software, La Jolla, CA, USA) software to analyse the data. Student’s *t*-test (two-tailed) or analysis of variance (ANOVA) test was used to compare tests under the assumptions of normality and the equality of variance. Otherwise, non-parametric tests (Mann–Whitney *U*-test or Kruskal–Wallis) were used. Categorical data were analysed by *χ*^2^ test or Fisher’s exact test. Kaplan–Meier analyses were used to assess the survival between subgroups (log-rank test). A Cox proportional hazards model was used to determine the independent factors of survival based on the variables selected in the univariate and multivariate analyses. The results are presented as mean ± standard deviation (SD). *p*-Values less than 0.05 were considered statistically significant.

## Results

### *Hsa-miR-27b* is downregulated in HCC tissues

**Fig. 1:**
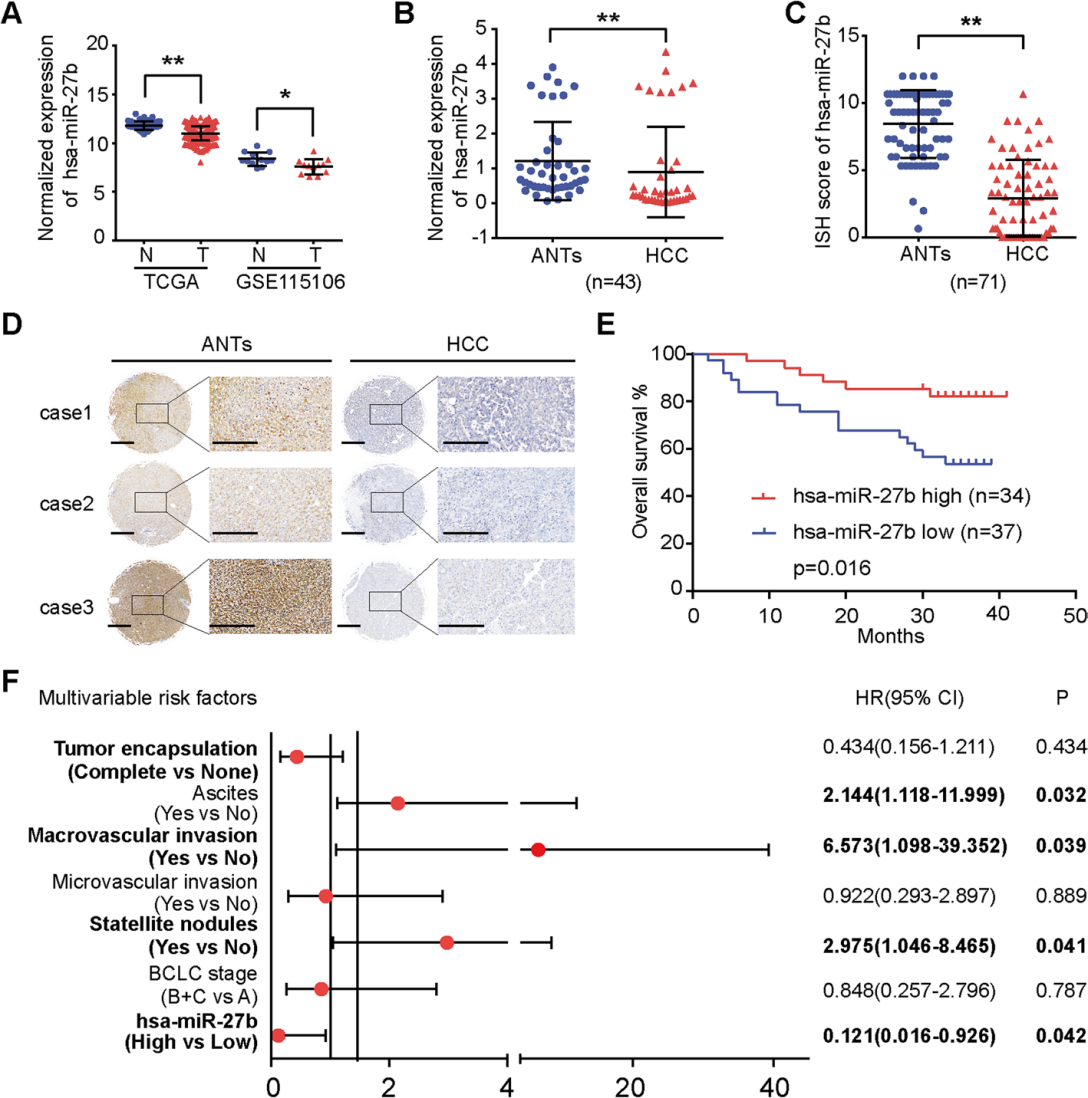
*Hsa-miR-27b* is downregulated in HCC specimens, and low expression of *hsa-miR-27b* predicts poor prognosis. **A** *Hsa-miR-27b* expression in HCC samples and normal liver tissues was analysed using TCGA (HCC, *n* = 370; normal liver, *n* = 50; unpaired *t*-test) and GSE115016 (HCC, *n* = 12; normal liver, *n* = 12; unpaired *t*-test) datasets. **B** qRT-PCR analysis of the expression of *hsa-miR-27b* in HCC and adjacent non-tumourous tissues (ANTs) (Mann–Whitney test). Data were normalized to *U6* and are shown as the fold change compared with patient 7. **C** ISH scoring of *hsa-miR-27b* in 71 pairs of HCC tissues (Mann–Whitney test). **D** Representative ISH images of *hsa-miR-27b* in paired HCC samples. Scale bar: overview images, 100 μm; magnified images, 200 μm. **E** Kaplan–Meier analysis of the correlation between *hsa-miR-27b* expression and overall survival in the cohort of patients with HCC (log-rank test). **F** Forest plot of the multivariate Cox proportional hazards model for overall survival. The bars correspond to 95% confidence intervals. *p < 0.05; **p < 0.01. Data are shown as mean ± SD. T, tumour; N, normal liver; ANTs, adjacent non-tumourous tissues; HCC, hepatocellular carcinoma; HR, hazard risk; CI, confidence interval

To investigate the role of *hsa-miR-27b* in HCC, we first analysed its expression level in cohorts of patients with HCC from the TCGA and the GEO (GSE115106) databases. The results showed that *hsa-miR-27*b expression was significantly decreased in HCC tissues compared with normal liver tissues (Fig. 1A). qRT-PCR analysis of 43 pairs of HCC samples verified the decreased expression of *hsa-miR-27b* in HCC compared with ANTs (Fig. 1B). ISH was then performed on 71 pairs of HCC specimens (Additional file 1: Table S3) to explore the relationship between *hsa-miR-27b* and the clinical features of patients with HCC. The ISH scores of *hsa-miR-27b* were significantly higher in ANTs than in counterpart HCC tissues (Fig. 1C, D and Additional file 1: Fig. S1A). Chi-squared analysis indicated that lower expression of *hsa-miR-27b* was significantly associated with higher serum α-fetoprotein (*AFP*) levels and advanced Child–Pugh grade (Additional file 1: Table S4). Kaplan–Meier analysis showed that patients with lower *hsa-miR-27b* levels exhibited shorter overall survival (OS) (Fig. 1E). In addition, univariate and multivariate Cox regression analyses demonstrated that lower expression of *hsa-miR-27b* was an independent risk factor for poor prognosis (Fig. 1F and Additional file 1: Table S5). Taken together, these results suggested that *hsa-miR-27b* was downregulated in HCC and that high expression of *hsa-miR-27b* predicted better prognosis.

### *Hsa-miR-27b* suppresses the proliferation, migration and invasion of HCC cells in vitro

**Fig. 2:**
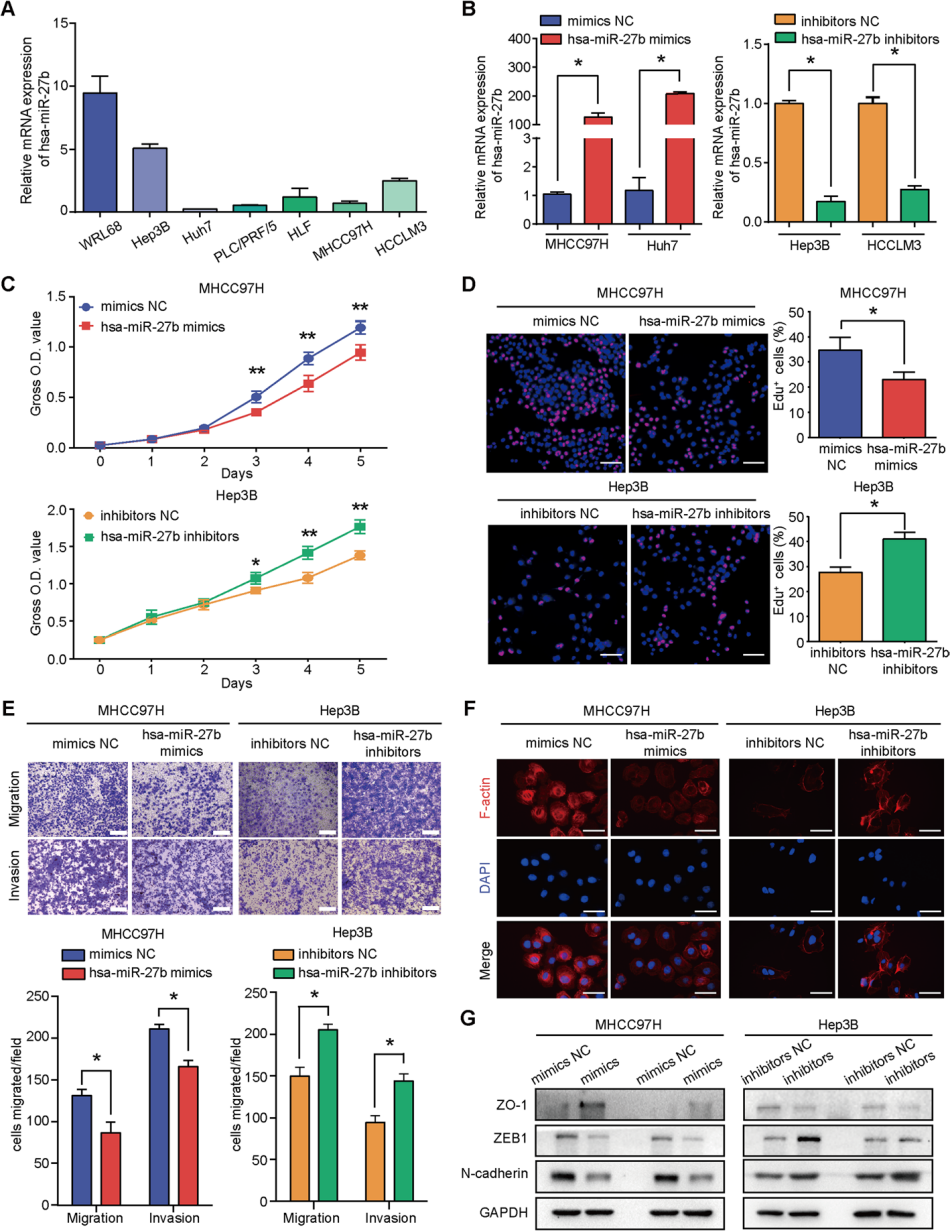
*Hsa-miR-27b* inhibits the proliferation and migration of HCC cells in vitro. **A** Endogenous expression of *hsa-miR-27b* in WRL68 and HCC cell lines. Data were normalized to *U6* and are shown as fold change compared with HLF cells (*n* = 3). **B** Overexpression or knockdown of *hsa-miR-27b* in HCC cells using *hsa-miR-27b* mimics or inhibitors, respectively, as measured by qRT-PCR. Data were normalized to *U6* and are shown as fold change relative to the control cells (*n* = 3, Mann–Whitney test). **C** The proliferation of the indicated cells with overexpression or knockdown of *hsa-miR-27b* as measured by CCK-8 assays (*n* = 5, Kruskal–Wallis test). **D** Representative images of the EdU incorporation assay (left). Scale bar: 100 μm; quantification of EdU-positive cells (right) (*n* = 3, Mann–Whitney test). **E** Representative images (top) and quantification (bottom) of cells that migrated or invaded in the indicated groups (*n* = 3, Mann–Whitney test). Scale bar, 200 μm. **F** Representative pictures of phalloidin staining for F-actin (red) in the indicated cells. DAPI was used to show the nucleus (blue). Scale bar, 20 μm. **G** Western blot analysis for EMT markers as indicated. *GAPDH* was used as a loading control. All experiments were repeated three times. *p < 0.05. **p < 0.01. Data are shown as mean ± SD. NC, negative control

We first determined *hsa-miR-27b* expression levels in the normal liver cell line WRL68 and six HCC cell lines by qRT-PCR (Fig. 2A). The results showed that the expression level of *hsa-miR-27b* was lower in the HCC cell lines than in WRL68. On the basis of the endogenous expression of *hsa*-*miR-27b* in HCC cell lines, we overexpressed *hsa-miR-27b* in MHCC97H and Huh7 cells with *hsa-miR-27b* mimics and knocked down *hsa-miR-27b* in Hep3B and HCCLM3 cells with *hsa-miR-27b* inhibitors (Fig. 2B). CCK-8 and EdU incorporation assays showed that *hsa-miR-27b* overexpression markedly inhibited and downregulation of *hsa-miR-27b* promoted proliferation of HCC cells (Fig. 2C, D and Additional file 1: Fig. S1B, C). To determine the effect of *hsa-miR-27b* on the migration and invasion of HCC cells, we carried out Transwell assays. Enhanced expression of *hsa-miR-27b* significantly impaired the migration and invasion capacities in MHCC97H and Huh7 cells, and vice versa in Hep3B and HCCLM3 cells (Fig. 2E and Additional file 1: Fig. S1D). Since epithelial–mesenchymal transition (EMT) is a key process in HCC metastasis [37], we first carried out fluorescence staining of *F-actin* in HCC cells with *hsa-miR-27b* manipulation. Overexpression of *hsa-miR-27b* inhibited actin cytoskeleton organization, while downregulation stimulated it (Fig. 2F and Additional file 1: Fig. S1E). Additionally, *hsa-miR-27b* increased the expression of epithelial markers (*ZO-1*) and decreased the expression of mesenchymal markers (*N-cadherin* and *ZEB1*), as evidenced by western blot analysis (Fig. 2G).

### *Hsa-miR-27b* inhibits tumour growth and lung metastasis in vivo

**Fig. 3:**
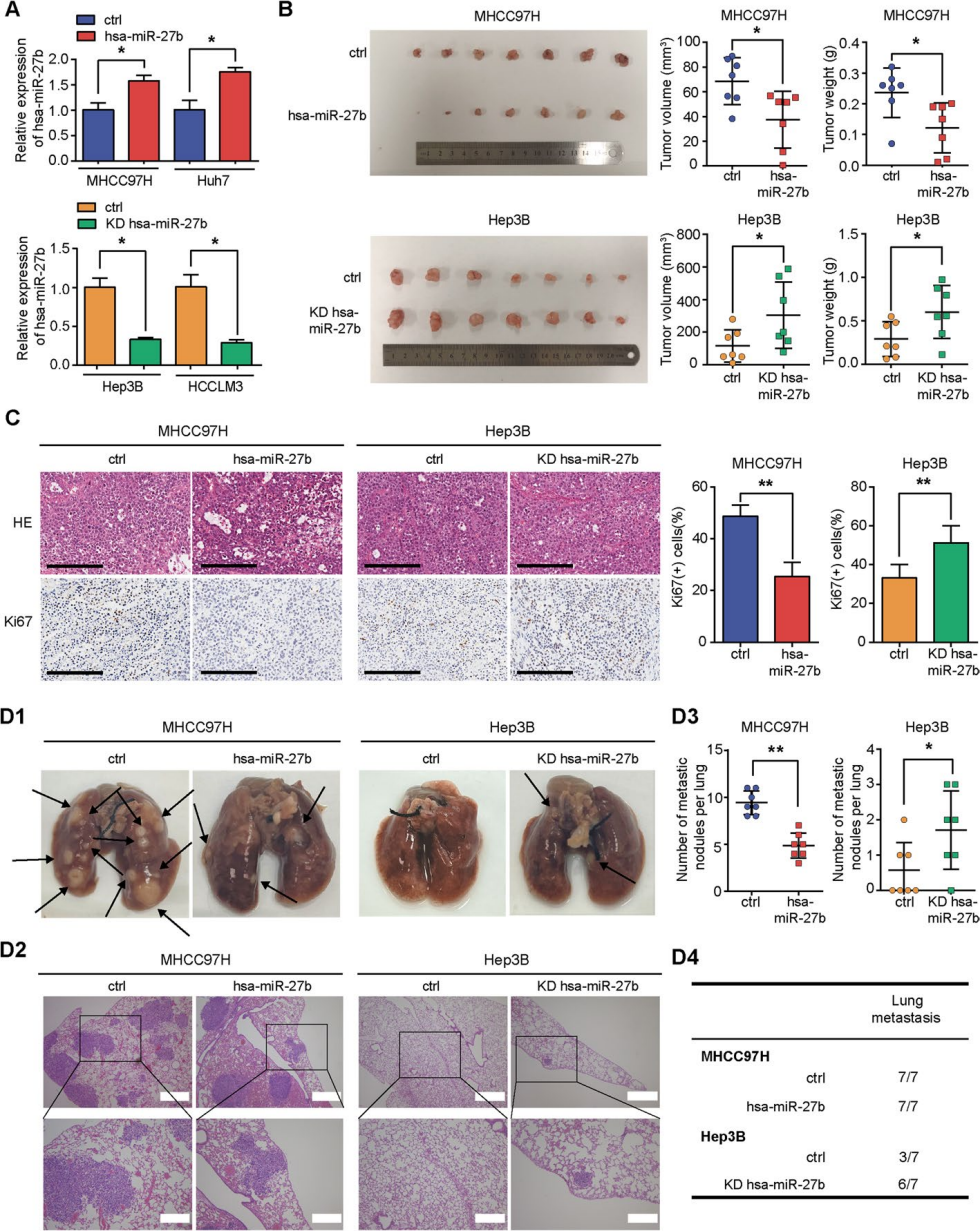
*Hsa-miR-27b* inhibits the growth and lung metastasis of HCC cells in vivo. **A** qRT-PCR analysis of *hsa-miR-27b* stable overexpression or knockdown efficacy in the indicated cells. Data were normalized to U6 and are shown as fold change relative to the control cells (*n* = 3, Mann–Whitney test). **B** Macroscopic images of subcutaneous tumours from mice (*n* = 7) bearing *hsa-miR-27b*-overexpressing MHCC97H cells or *hsa-miR-27b*-knockdown Hep3B cells (left); quantification of the volumes and weights of subcutaneous tumours in the indicated groups (right); unpaired *t*-test. **C** Representative haematoxylin and eosin (H&E) staining and IHC of Ki67 in subcutaneous xenografts. Quantification of staining scores was calculated (*n* = 7, unpaired *t*-test); Scale bar, 200 μm. **D** Lung metastasis experiments were performed in nude mice with the indicated cells. **D1** and **D2** Representative macroscopic and H&E staining images of lung metastases. **D3** Quantification of visible tumour nodules. MHCC97H: control group (*n* = 7); hsa-miR-27b group (*n* = 7); unpaired *t*-test. Hep3B: control group (*n* = 7); KD *hsa-miR-27b* group (*n* = 7); Mann–Whitney test. **D4** Lung metastatic incidence in different groups; *p < 0.05. **p < 0.01. Data are shown as mean ± SD. ctrl, control; KD, knockdown

We stably overexpressed *hsa-miR-27b* in MHCC97H and Huh7 cells (MHCC97H/*hsa-miR-27b*, Huh7/*hsa-miR-27b*) and knocked down *hsa-miR-27b* in Hep3B and HCCLM3 cells (Hep3B/KD *hsa-miR-27b*, HCCLM3/KD *hsa-miR-27b*) by lentivirus (Fig. 3A). HCC cells were subcutaneously injected into nude mice to investigate the effects of *hsa-miR-27b* on tumourigenicity in vivo. Thirty days after inoculation, the mice were sacrificed to assess tumour growth. Tumour volumes and weights were significantly lower in the mice injected with the *hsa-miR-27b*-overexpressing MHCC97H cells than in the mice injected with the control cells. The opposite results were observed in the *hsa-miR-27b*-knockdown Hep3B cells (Fig. 3B). IHC assays of Ki-67 showed that tumour cells with higher *hsa-miR-27b* levels exhibited weaker proliferation than their corresponding controls (Fig. 3C). In vivo tumour lung metastasis assays showed that the mice bearing MHCC97H cells with *hsa-miR-27b* overexpression developed fewer metastatic nodules than the mice in the control group. Consistently, knocking down *hsa-miR-27b* in Hep3B enhanced its metastatic ability, as evidenced by a higher incidence of lung metastasis and more metastatic nodules than in the control group (Fig. 3D).

### *TAB3* is the downstream target of *hsa-miR-27b*

**Fig. 4:**
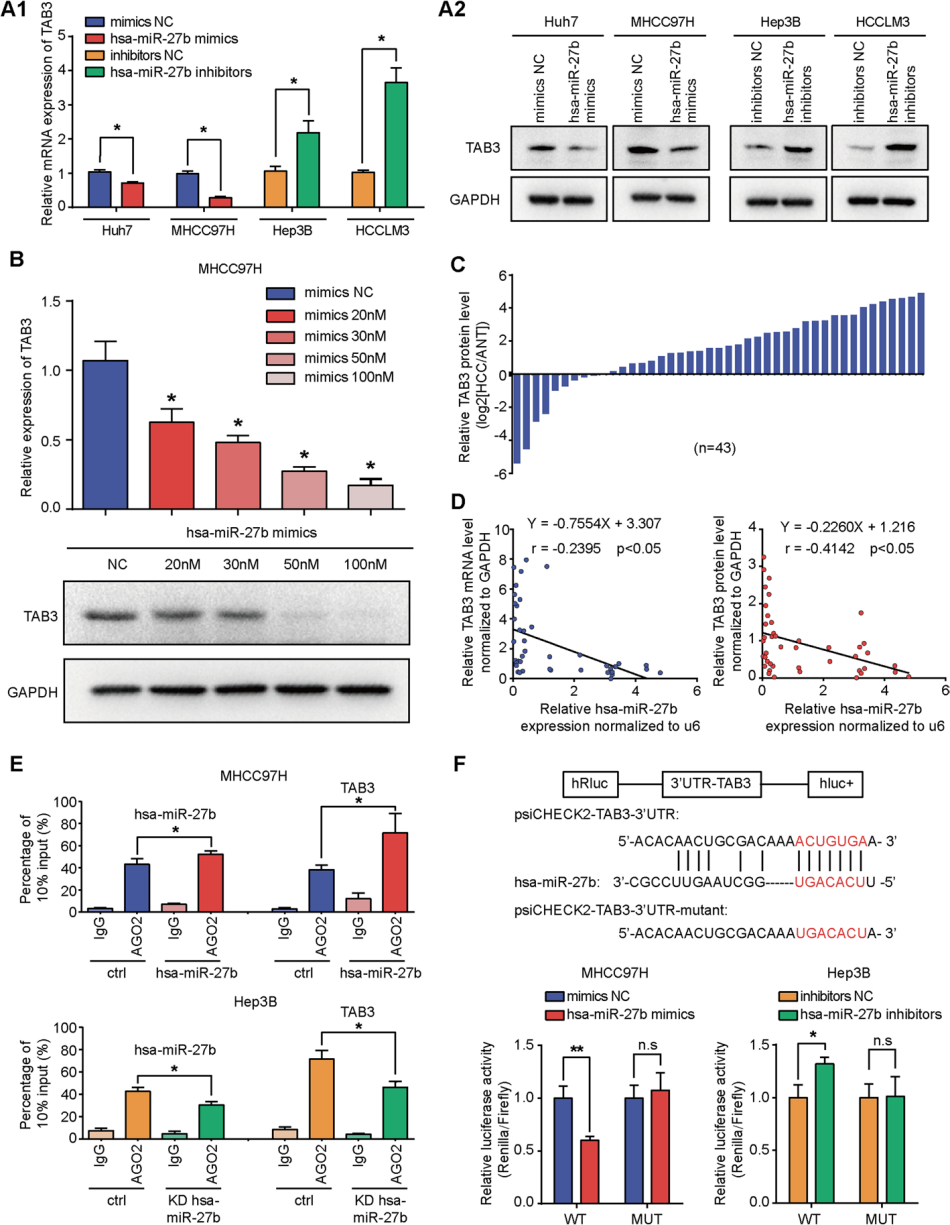
*TAB3* is the downstream target of *hsa-miR-27b*. **A1**, **A2** qRT-PCR (**A1**) and western blot (**A2**) analysis of *TAB3* mRNA and protein levels after overexpression or knockdown of *hsa-miR-27b *in HCC cells. Data were normalized with *GAPDH* and are shown as the fold change relative to the control cells. *GAPDH* was used as a loading control for western blot analysis (*n* = 3 for qRT-PCR, Mann–Whitney test). **B** MHCC97H cells were transfected with *hsa-miR-27b* mimics at the indicated concentrations. qRT-PCR analysis of *TAB3* mRNA (top) and western blot analysis of *TAB3* protein levels (bottom) were performed. Data were normalized to *GAPDH* and are shown as fold change compared with the cells treated with an NC for qRT-PCR (*n* = 3, one-way ANOVA). *GAPDH* was used as a loading control for western blot analysis. **C** Relative *TAB3* expression in HCC tissues (*n* = 43) compared with ANTs (*n* = 43) analysed by western blots. **D** Linear regression analysis between relative *TAB3* mRNA (left) or protein levels (right) and *hsa-miR-27b* expression in HCC specimens (*n* = 43). **E** RNA immunoprecipitation assays of 3′UTR-*TAB3* enrichments by anti-IgG or anti-*AGO2* antibodies in the *hsa-miR-27b*-overexpressing MHCC97H cells (top) or the *hsa-miR-27b*-knockdown Hep3B cells (bottom). Data are shown as percentage of 10% input (*n* = 3, Kruskal–Wallis test). **F** Schematic diagram of *hsa-miR-27b* binding sequences in the 3′UTR of *TAB3* and mutants in the 3′UTR of *TAB3* (*TAB3*-MUT) (top). Dual luciferase reporter assay of MHCC97H and Hep3B cells co-transfected with wild-type (WT) or mutated *TAB3* 3′UTR luciferase reporter plasmids with *hsa-miR-27b* mimics or inhibitors (*n* = 3, Mann–Whitney test). **A**, **B**, **E** and **F** were repeated three times. *p < 0.05. **p < 0.01. Data are shown as mean ± SD. NC, negative control; ANT: adjacent non-tumourous tissues; HCC, hepatocellular carcinoma; 3′UTR, 3′ untranslated region; ctrl, control; KD, knockdown

To explore the downstream effectors of *hsa-miR-27b*, we generated a Venn diagram to overlap the predicted *hsa-miR-27b* targets from three independent websites: miRDB, miRWalk and TargetScan (Additional file 1: Fig. S2A). The top ten predicted targets were selected for qRT-PCR verification (Fig. 4A1 and Additional file 1: Fig. S2B). The mRNA and protein levels of *TAB3*, which is involved in the activation of *NF-κB* signalling, were downregulated by *hsa-miR-27b* overexpression and upregulated by *hsa-miR-27b* knockdown (Fig. 4A). Moreover, the mRNA and protein levels of *TAB3* decreased coordinately with increasing doses of the *hsa-miR-27b* mimics in MHCC97H cells (Fig. 4B). The expression of *TAB3* in tumours was significantly higher than that in non-tumourous liver tissue, as shown by western blot (Fig. 4C and Additional file 1: Fig. S2C). Additionally, the *hsa-miR-27b* level was negatively correlated with the mRNA (*r* = −0.2395, *p* < 0.05) and protein levels of *TAB3* (*r* = −0.4124, *p* < 0.05) (Fig. 4D). IHC analysis of subcutaneous tumour sections from nude mice showed that the expression level of *TAB3* increased in the *hsa-miR-27b* knockdown group and decreased in the *hsa-miR-27b* overexpression group (Additional file 1: Fig. S2D). *AGO2* was initially reported as the catalytic centre of the RNA-induced silencing complex [38]. Thus, we conducted an anti-*AGO2* RIP assay. The results showed that *hsa-miR-27b* and *TAB3* mRNA were enriched in anti-*AGO2* precipitate. Moreover, the abundance of *TAB3* mRNA in anti-*AGO2* precipitate increased in the *hsa-miR-27b*-overexpressing MHCC97H cells and decreased in the *hsa-miR-27b*-knockdown Hep3B cells (Fig. 4E). The potential binding site of *hsa-miR-27b* in the *TAB3* 3′UTR was predicted by miRBase (Fig. 4F), and a dual luciferase reporter assay showed that the relative luciferase activity of *TAB3*-3′UTR was decreased in *hsa-miR-27b*-overexpressing MHCC97H and Huh7 cells, but increased in *hsa-miR-27b*-knockdown Hep3B and HCCLM3 cells. However, co-transfection of *TAB3*-MUT and the *hsa-miR-27b* mimics or inhibitors had no such effect (Fig. 4F and Additional file 1: Fig. S2E). These results demonstrated that *hsa-miR-27b* bound to the 3′UTR of *TAB3* mRNA and decreased its expression in an *AGO2*-dependent manner.

### *TAB3* mediates the impacts of *hsa-miR-27b* in HCC

**Fig. 5:**
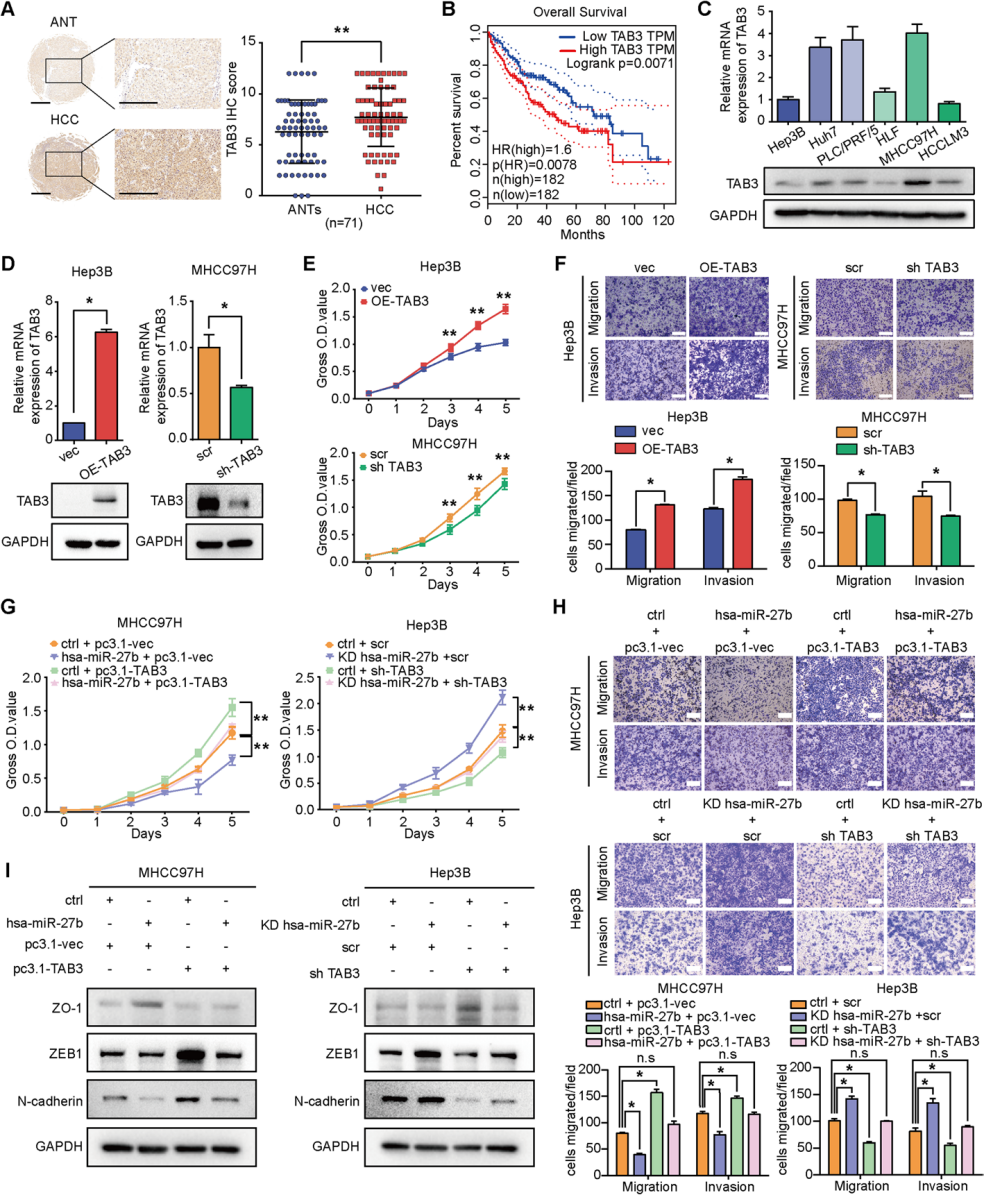
The impacts of *hsa-miR-27b* on HCC cells were mediated by *TAB3*. **A** Representative images (left) and quantification (unpaired *t*-test) (right) of *TAB3* IHC staining in 71 pairs of HCC tissues and ANTs. Scale bar: left, 100 μm; right, 200 μm. **B** Kaplan–Meier analysis of the relationship between *TAB3* expression and overall survival of patients with HCC in the GEPIA database (log-rank test). **C** *TAB3* mRNA and protein expression in HCC cell lines as determined by qRT-PCR (top) and western blots (bottom). Data were normalized to *GAPDH* and are shown as fold change compared with Hep3B cells for qRT-PCR (*n* = 3). *GAPDH* was used as a loading control for western blot analysis. **D** qRT-PCR (top) and western blot (bottom) analysis of *TAB3* overexpression or knockdown efficacy in the indicated cells. Data were normalized to *GAPDH* and are shown as fold change relative to their respective control cells for qRT-PCR (*n* = 3, Mann–Whitney test). *GAPDH* was used as a loading control for western blot analysis. **E** CCK-8 assay of the indicated cells (*n* = 5, Kruskal–Wallis test). **F** Representative images of cell migration and invasion in the indicated groups (top). Scale bar, 200 μm. Quantification of cell migration and invasion (*n* = 3, Mann–Whitney test) (bottom). **G** CCK-8 assay for the indicated cell groups (*n* = 5, Kruskal–Wallis test). **H** Representative images and quantification of migrated and invaded cells in the indicated cell groups (*n* = 3, Mann–Whitney test). Scale bar, 200 μm. **I** Western blotting for EMT markers as indicated. *GAPDH* was used as a loading control. **C**–**I** were repeated three times. *p < 0.05. **p < 0.01. Data are shown as mean ± SD. ANTs, adjacent non-tumourous tissues; HCC, hepatocellular carcinoma; vec, vector; OE, overexpression; scr, scramble; sh, small hairpin RNA; ctrl, control; vec, vector; pc3.1-, pcDNA3.1-; KD, knockdown; sh, small hairpin RNA

IHC analysis of *TAB3* in paired clinical HCC specimens was performed to analyse the clinical relevance of *TAB3*. The average scoring of *TAB3* was significantly higher in HCC than in its corresponding ANTs (Fig. 5A and Additional file 1: Fig. S3A). Kaplan–Meier survival analysis from the GEPIA database showed that patients with HCC with higher expression of *TAB3* protein had significantly shorter OS times than patients with HCC with lower *TAB3* expression (Fig. 5B). To investigate the role of *TAB3* in HCC, we first analysed its expression level in the HCC cell line (Fig. 5C). We then used lentivirus to stably knock down *TAB3* in MHCC97H and Huh7 cells with relatively low endogenous *hsa-miR-27b* expression, and overexpressed *TAB3* in HCCLM3 and Hep3B cells with relatively high endogenous *hsa-miR-27b* expression (Fig. 5D and Additional file 1: Fig. S3B). The CCK-8 assay showed that downregulation of *TAB3* inhibited, and overexpression of *TAB3* promoted, cell proliferation in HCC cells (Fig. 5E and Additional file 1: Fig. S3C). Transwell assays showed that cells with higher *TAB3* expression levels displayed stronger migration and invasion than those with lower *TAB3* expression levels (Fig. 5F and Additional file 1: Fig. S3D).

To confirm whether *TAB3* is critically involved in *hsa-miR-27b*-mediated cell growth, migration and invasion, we enhanced *TAB3* expression in the *hsa-miR-27b*-overexpressing MHCC97H cells, or knocked down *TAB3* in the *hsa-miR-27b*-knockdown Hep3B cells. The impaired proliferation, migration and invasion induced by *hsa-miR-27b* overexpression were recovered by overexpressing *TAB3* in MHCC97H cells, and vice versa in the *hsa-miR-27b*-knockdown Hep3B cells with *TAB3* downregulation (Fig. 5G–I).

### *Hsa-miR-27b* inhibits *NF-кB* activity by targeting *TAB3* in HCC cells

**Fig. 6:**
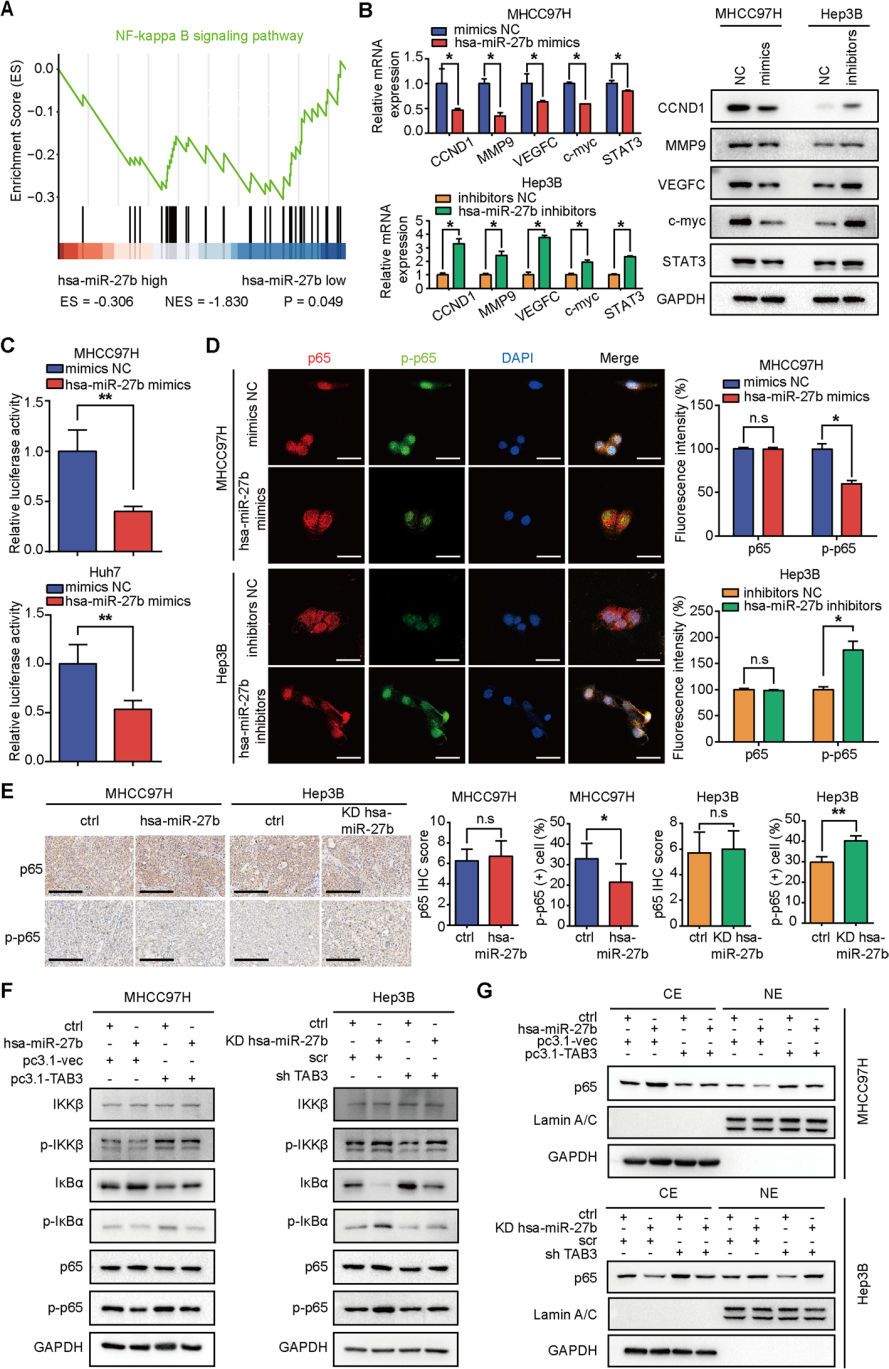
*Hsa-miR-27b* inhibits *NF-кB* signalling activity by targeting *TAB3* in HCC cells. **A** GSEA of the correlation between *hsa-miR-27b* and *NF-кB* target gene signatures using the cohort of patients with HCC in the TCGA database. **B** *NF-кB* target gene mRNA (left) and protein (right) levels in the indicated cells. Data were normalized to *GAPDH* and are shown as fold change relative to the control cells for qRT-PCR (*n* = 3, Mann–Whitney test). *GAPDH* was used as a loading control for western blotting. **C** Dual luciferase reporter analysis for *NF-кB* transcriptional activity in the indicated cells. Data were normalized to the NC cells (*n* = 3, Mann–Whitney test). **D** Confocal images of immunofluorescence staining of *p65* and p-*p65* in the indicated cells. Scale bar, 200 μm (*n* = 3, Mann–Whitney test). **E** Representative IHC images and quantification of IHC scoring for *p65* and phospho-*p65* in subcutaneous tumours (*n* = 7, unpaired *t*-test). Scale bar, 200 μm. **F** Western blot analysis of protein expression in the indicated cells treated with *TNF-α* (10 ng/ml). *GAPDH* was used as a loading control. **G** Western blot analysis of *p65* expression in the nucleic/cytoplasmic fractions after treatment with *TNF-α* (10 ng/ml) in the indicated cells. *GAPDH* or *lamin A/C* was used as a positive control for the cytoplasmic or nucleic fraction. **B**–**G** were repeated three times. *p < 0.05. **p < 0.01. Data are shown as mean ± SD. ES, enrichment score; NES, normalized enrichment score; NC, negative control; KD, knockdown; ctrl, control. CE, cytoplasmic extracts; NE, nuclear extracts

Gene set enrichment analysis (GSEA) for the patient cohort from TCGA showed that the level of *hsa-miR-27b* was inversely correlated with the *NF-кB*-activated gene signatures, suggesting that *hsa-miR-27b* might be involved in the de-activation of *NF-кB* signalling, which was consistent with its negative regulation of *TAB3* expression (Fig. 6A). Furthermore, cyclin D1 (*CCND1*), matrix metallopeptidase 9 (*MMP9*), vascular endothelial growth factor C (*VEGFC*), Myc proto-oncogene (*c-myc*) and signal transducer and activator of transcription 3 (*STAT3*), the downstream effectors of *NF-кB* signalling [39], were found to be negatively regulated by *hsa-miR-27b*, implying that *hsa-miR-27b* downregulation contributed to *NF-кB* activation in HCC cells (Fig. 6B). A dual-luciferase reporter assay showed that overexpression of *hsa-miR-27b* reduced *NF-кB*-induced luciferase activity in MHCC97H and Huh7 cells (Fig. 6C). Immunofluorescence staining showed that overexpressing *hsa-miR-27b* led to a significant cytoplasmic location of *p65* and weaker fluorescence intensity of *p-p65*, whereas silencing *hsa-miR-27b* resulted in *p65* accumulation in the nucleus and stronger signals for p-*p65* (Fig. 6D). IHC analysis of subcutaneous tumour sections from nude mice showed that the expression level of p-*p65* increased in the low *hsa-miR-27b* expression group and decreased in the high *hsa-miR-27b* expression group (Fig. 6E). Collectively, these results suggested that *hsa-miR-27b* inhibited *NF-кB* signalling in HCC.

Co-IP assay demonstrated that *TAB3* bound with *TAK1* in HCC cells (Additional file 1: Fig. S3E), which was consistent with previous reports [40]. Additionally, impairing the expression of *TAK1* in *TAB3*-overexpressed HCC cells impeded the enhanced *NF-кB* activity evidenced by dual luciferase reporter assays (Additional file 1: Fig. S3F, G). These results indicated that the impact of *TAB3* on NF-кB signalling was mediated by *TAK1*. To determine whether *TAB3* mediated the modulation of *NF-кB* signalling by *hsa-miR-27b*, we overexpressed *TAB3* in the *hsa-miR-27b*-overexpressing MHCC97H cells and knocked down *TAB3* in the *hsa-miR-27b*-knockdown Hep3B cells. The impaired p-*IKKβ*, p-*IκBα* and p-*p65* levels induced by *hsa-miR-27b* overexpression were recovered by ectopic expression of *TAB3* in MHCC97H cells, while the enhanced *IκBα* expression was also restored. Consistent results were obtained in the *hsa-miR-27b* knockdown cells with *TAB3* downregulation (Fig. 6F). Additionally, the decreased/elevated nuclear location of *p65* in the *hsa-miR-27b*-overexpressing/knockdown HCC cells was rescued by overexpressing/knocking down *TAB3* (Fig. 6G). Taken together, these results demonstrated that decreased expression of *TAB3* mediated the de-activation of *NF-κB* signalling by *hsa-miR-27b*, and the *TAB3*/*IKKβ*/*IκBα* signalling axis was modulated by *hsa-miR-27b*.

### Onco^Ad^*hsa-miR-27b* inhibits HCC growth

**Fig. 7:**
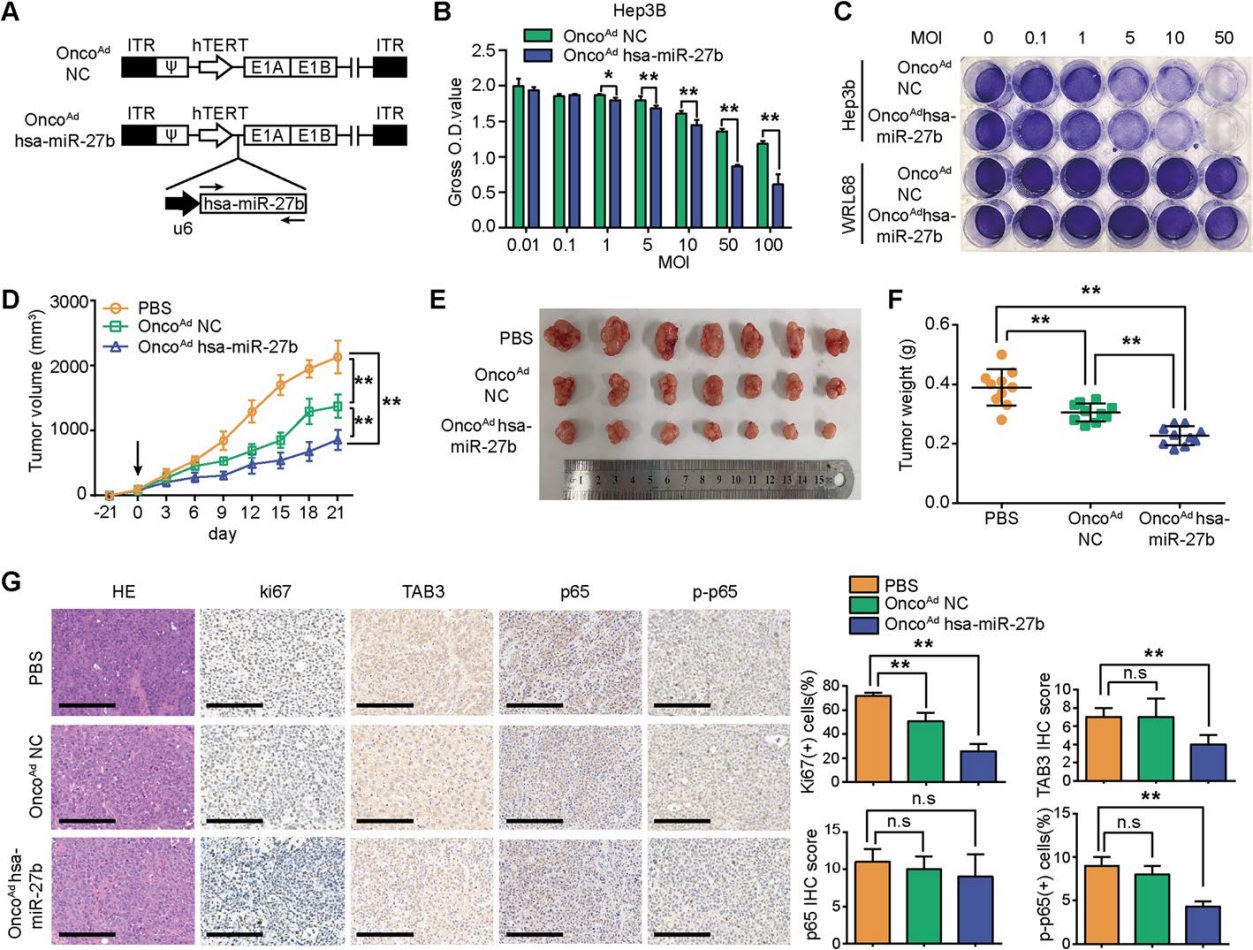
Onco^Ad^ *hsa-miR-27b* suppressed HCC growth in vitro and in vivo. **A** Schematic diagram of the structures of the Onco^Ad^. **B** Hep3B cells were treated with Onco^Ad^ *hsa-miR-27b* and Onco^Ad^ NC at the indicated MOI. After 48 h, cell viabilities were measured by CCK-8 assays (*n* = 5, unpaired *t*-test). **C** Hep3B and WRL68 cells were infected with Onco^Ad^ at the indicated MOIs for 2 days. Images of cells stained with crystal violet indicated the cytopathic effect of Onco^Ad^ *hsa-miR-27b* and Onco^Ad^ NC. **D** Growth curve of subcutaneous tumours with intra-tumoural injection of PBS (*n* = 7), Onco^Ad^ NC (*n* = 7) or Onco^Ad^ *hsa-miR-27b* (*n* = 7). Onco^Ad^ were intra-tumourally injected every 2 days after inoculating subcutaneous tumours for 21 days (Day 0); PBS was injected as a control. The arrow represents the first action of intra-tumoural injection, two-way ANOVA. **E** and **F** Macroscopic images and quantification of tumour weights of the Hep3B xenografts treated with PBS (*n* = 7), Onco^Ad^ NC (*n* = 7) or Onco^Ad^ *hsa-miR-27b* (*n* = 7) at 21 days post-intra-tumoural injection, one-way ANOVA. **G** Representative images of H&E staining and IHC staining of the indicated protein in Hep3B xenografts; scale bar, 200 μm. Quantification of Ki-67 and p-*p65*-positive cells, as well as IHC scoring of *TAB3* and *p65* (*n* = 7, one-way ANOVA). **B** and **C** were repeated three times. *p < 0.05, **p < 0.01. Data are shown as mean ± SD. NC, negative control; Onco^Ad^, oncolytic adenovirus. MOI, multiplicities of infection; ITR, inverted terminal repeat

Onco^Ad^ represents a promising therapeutic approach that exhibits anti-tumour effects through selective tumour cell killing [41]. Herein, we constructed an Onco^Ad^ overexpressing *hsa-miR-27b* with the *U6* promoter (Fig. 7A). The replication of Onco^Ad^ measured by qRT-PCR demonstrated that they selectively replicated in tumour cells (Additional file 1: Fig. S4A). Onco^Ad^ *hsa-miR-27b* efficiently overexpressed *hsa-miR-27b* in WRL68 and Hep3B cells (Additional file 1: Fig. S4B). To evaluate the HCC-specific cytotoxicity of Onco^Ad^, we infected hepatoma Hep3B cells and liver WRL68 cells with Onco^Ad^ *hsa-miR-27b* or the control virus Onco^Ad^ NC at the indicated MOIs. A cytopathic effect was observed in the Hep3B cells infected with Onco^Ad^ *hsa-miR-27b*, and the cytopathic effect of Onco^Ad^ *hsa-miR-27b* was superior to that of Onco^Ad^ NC, while no obvious cytotoxicity was observed in WRL68 cells at the same MOIs (Fig. 7B, C). In addition, the CCK-8 assay indicated that Onco^Ad^ *hsa-miR-27b* showed higher cytotoxicity than Onco^Ad^ NC at the same MOI in a time-dependent manner (Additional file 1: Fig. S4C). Moreover, the proportion of cracked nuclei exhibited by Hoechst 33342 staining was significantly increased in the Onco^Ad^ *hsa-miR-27b*-treated Hep3B cells compared with the Onco^Ad^ NC-treated cells, while no such effect was observed in the Onco^Ad^ *hsa-miR-27b-* or Onco^Ad^ NC-treated WRL68 cells (Additional file 1: Fig. S4D).

The in vivo anti-tumour efficacy of Onco^Ad^ *hsa-miR-27b* was evaluated in a Hep3B xenograft animal model. The average volumes and weights of subcutaneous tumours were significantly lower in the mice with administration of Onco^Ad^ *hsa-miR-27b* than in the mice administered PBS or Onco^Ad^ NC (Fig. 7D–F). In addition, Onco^Ad^ *hsa-miR-27b* succeeded in downregulating *TAB3* and p-*p65* expression levels in Hep3B xenografts. Moreover, the Onco^Ad^ *hsa-miR-27b*-treated tumours showed weaker proliferation measured by Ki67 staining than that in the other groups (Fig. 7G). These results demonstrated the anti-tumoural potential of Onco^Ad^ *hsa-miR-27b* in HCC.

## Discussion

Recent studies have reported that *hsa-miR-27b* is involved in tumour progression through multiple pathways. The functions of *hsa-miR-27b* in cancer development are highly contextual [42]. Jin et al. showed that *hsa-miR-27b* was highly upregulated in human breast cancer, and knockdown of *hsa-miR-27b* repressed breast cancer growth [10]. Inconsistently, Tao et al. reported that *hsa-miR-27b* suppressed gastric cancer cell proliferation and colony formation [43]. In HCC, the current views of the role of *hsa-miR-27b* are contradictory [14–16]. A copy number variation analysis of TCGA database showed that the *hsa-miR-27b* gene locus was lost in 12 of 49 liver cancer samples, and the expression of *hsa-miR-27b* was found to be downregulated compared with that in normal liver tissues in TCGA and ArrayExpress databases [17]. The decreased expression of *hsa-miR-27b* in HCC was further confirmed using qRT-PCR by two independent research groups [16, 17]. By analysing the TCGA database (372 HCC tissues versus 50 normal adjacent liver tissues) and using qRT-PCR assay of 68 pairs of HCC specimens (patients with hepatitis B virus and without metastases), Liang et al. also determined that the level of *hsa-miR-27b* were decreased in HCC. Besides, Fu et al. showed that *hsa-miR-27b* was downregulated at mature miRNA level but upregulated at premature level (66 pairs of tissue samples) [18]. However, opposite results were obtained by Sun et al. [14] and He et al. [15]. qRT-PCR analysis of 90 and 35 pairs of HCC patient specimens demonstrated that the expression of *hsa-miR-27b* was higher in HCC tissues than in their corresponding normal liver tissues [14, 15]. In our study, we comprehensively analysed the expression pattern of *hsa-miR-27b* using bioinformatic analysis (two datasets), qRT-PCR (*n* = 43) and ISH examinations (*n* = 71). We concluded that the expression of *hsa-miR-27b* was downregulated in tumour tissues and that lower expression of *hsa-miR-27*b in HCC was an independent risk factor for shorter OS.

Multiple researchers have documented that the *NF-кB* signalling pathway is constitutively activated and may act as a promising target for the treatment of HCC [23, 44]. *TAB3* is involved in regulating differentiation and progression in several cancer types by modulating *NF-кB* signalling [27, 29, 30, 45]. In HCC, *hsa-miR-195* suppressed cell proliferation and migration in vitro and in vivo by decreasing the expression of multiple *NF-κB* downstream effectors via direct targeting of *IKKα* and *TAB3* [46]. Zhao et al. showed that *hsa-miR-26b* suppresses *NF-κB* signalling and thereby sensitizes HCC cells to doxorubicin-induced apoptosis by binding the 3′UTR of *TAK1* and *TAB3* [25]. In addition, *hsa-miR-195*, *hsa-miR-342-3p* and *hsa-miR-26b* target the *NF-κB* pathway by binding to the 3′UTR of *TAB3* [25, 46, 47].

Although miRNA-mediated *NF-кB* signalling inhibition is a promising cancer treatment method, miRNAs as cancer therapeutics need to overcome the problems of instability. As new technologies emerging in the construction of miRNAs, their mimetics, and delivery vehicles, miRNAs are becoming more viable for many cancer treatments, especially in chemo- and radiotherapy combinations [48]. Prior studies have noted that Onco^Ad^ may provide a promising platform for miRNA-based cancer therapy [41]. Onco^Ad^ co-expressing *hsa-miR-34a* and interleukin-24 (*IL-24*) induced increased anti-tumour activity compared with Onco^Ad^ expressing *hsa-miR-34a* or *IL-24* alone [49]. Callegari et al. developed a novel Onco^Ad^, Ad-199T, which could replicate more efficiently in tumour tissues in an *hsa-miR-199*-dependent manner in HCC HepG2 cells [50]. In this study, we constructed Onco^Ad^ *hsa-miR-27b* to deliver *hsa-miR-27b* for the treatment of HCC. A cytopathic effect was observed in the Hep3B cells infected with Onco^Ad^ *hsa-miR-27b*, and little cytotoxicity was observed in normal liver cells.

## Conclusions

In this study, we demonstrated that *hsa-miR-27b* was a tumour suppressor in HCC. *Hsa-miR-27b* suppressed the proliferation, migration and invasion of cancer cells to inhibit HCC growth and metastasis. Besides, *TAB3* was identified as the target downstream effector of *hsa-miR-27b*. Moreover, we demonstrated that the inactivation of *NF-кB* signalling by *hsa-miR-27b* was mediated by *TAB3* through the *IKKβ*/*IκBα* axis. Compared with Onco^Ad^ NC, Onco^Ad^ *hsa-miR-27b* induced stronger anti-tumour activity in vivo and in vitro. Therefore, our results implied that *hsa-miR-27b* could serve as a potential diagnostic and therapeutic target for HCC.

## Supplementary Information

**Additional file 1.** Additional Figures S1–S4 and Tables S1–S5.

## Data Availability

Datasets used and/or analysed in this study can be obtained from the corresponding author on reasonable request.

## Abbreviations

*AFP*: α-Fetoprotein
ANTs: Adjacent normal tissues
*AGO2*: Argonaute 2
CCK-8: Cell Counting Kit-8
*Bcl-2*: BCL2 apoptosis regulator
*CCND1*: Cyclin D1
*c-myc*: Myc proto-oncogene
co-IP: Co-immunoprecipitation
Edu: 5-Ethynyl-2′-deoxyuridine
EMT: Epithelial–mesenchymal transition
*F-actin*: Filamentous actin
GEO: Gene Expression Omnibus
GSEA: Gene set enrichment analysis
HCC: Hepatocellular carcinoma
IHC: Immunohistochemistry
*IKB-α*: I-kappa-B-alpha
*IKK*: Inhibitor of NF-кB kinase
*IL-24*: Interleukin-24
ISH: In situ hybridization
ITR: Inverted terminal repeat
LIHC: Liver hepatocellular carcinoma
miRNA: MicroRNA
*Hsa-miR-27b*: Hsa-microRNA-27b-3p
*MMP9*: Matrix metallopeptidase 9
mRNA: Messenger RNA
MOI: Multiplicities of infection
NC: Negative control
*NF-кB*: Nuclear factor kappa-B
O.D.: Optical density
Onco^Ad^*hsa-miR-27b*: Oncolytic adenovirus with overexpression of *hsa-miR-27b*
Onco^Ad^ NC: Oncolytic adenovirus with negative control
OS: Overall survival
*p65*: RELA proto-oncogene
PBS: Phosphate-buffered saline
p-*IKKβ*: Phospho-inhibitor kappa B kinase-β
qRT-PCR: Quantitative real-time PCR
RIP: RNA immunoprecipitation
*STAT3*: Signal transducer and activator of transcription 3
*TAK1*: Transforming growth factor β-activated protein kinase 1
*TAB2*: Transforming growth factor-activated kinase-binding protein 2
*TAB3*: Transforming growth factor-activated kinase (*TAK1*)-binding protein 3
TCGA: The Cancer Genome Atlas
*TNF-α*: Tumour necrosis factor α
TPM: Transcripts per million
*VEGFC*: Vascular endothelial growth factor C
*ZEB1*: Zinc finger E-box binding homeobox 1
*ZO-1*: Tight junction protein 1
3ʹUTR: 3′-Untranslated region
