# Supplementary material for: Hsa-microRNA-27b-3p inhibits hepatocellular carcinoma progression by inactivating transforming growth factor-activated kinase-binding protein 3/nuclear factor kappa B signalling
Source: Cell Mol Biol Lett. 2022 Sep 23;27:79. doi: 10.1186/s11658-022-00370-4 (PMC9502615; doi:10.1186/s11658-022-00370-4)
Supplement: Supplementary file 1 — Additional file 1. Additional Figures S1–S4 and Tables S1–S5. [file 11658_2022_370_MOESM1_ESM.docx]

**Additional information:**

**Additional figures:**

**Figure S1**

**
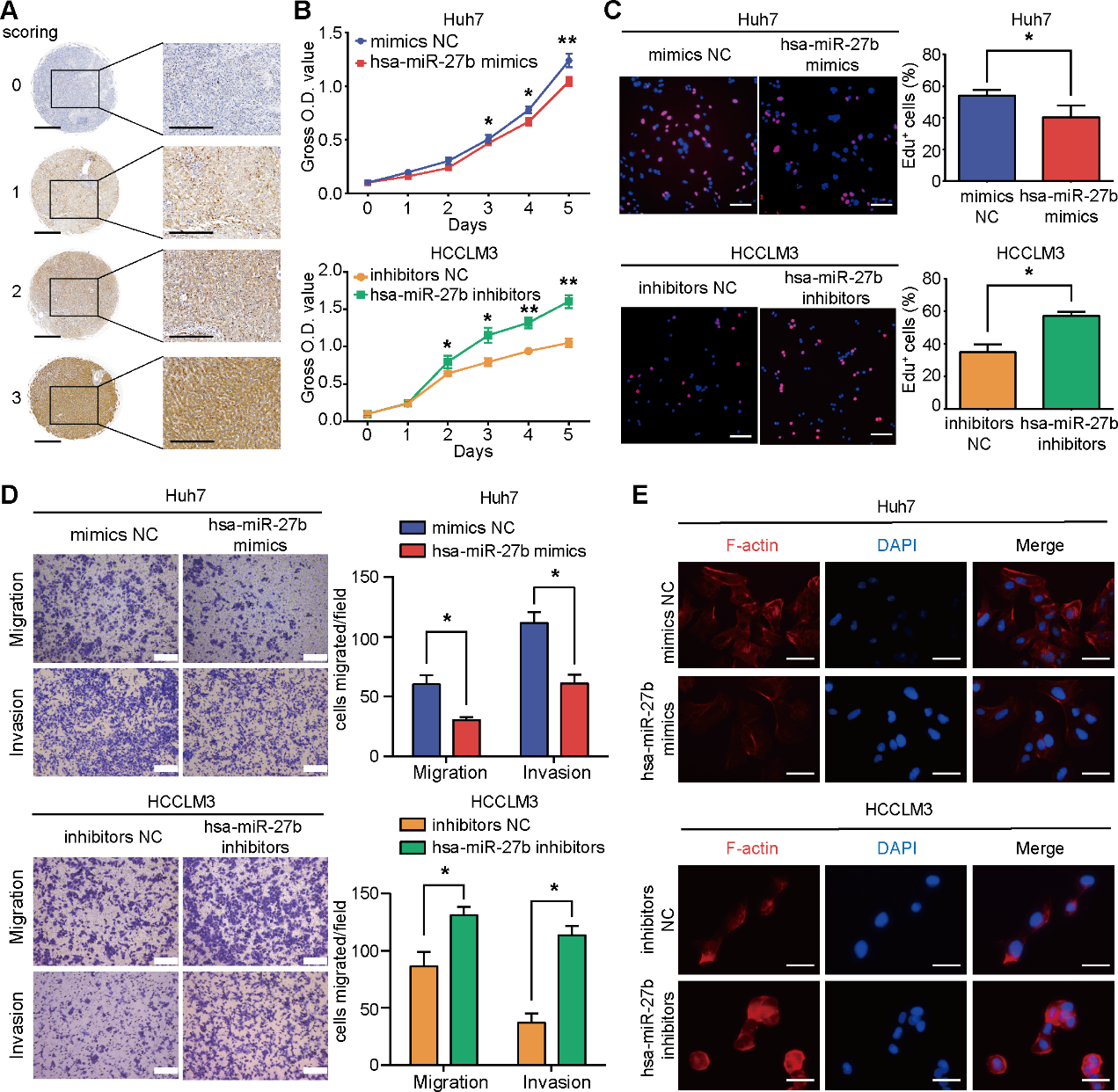
**

**Figure S2**

**
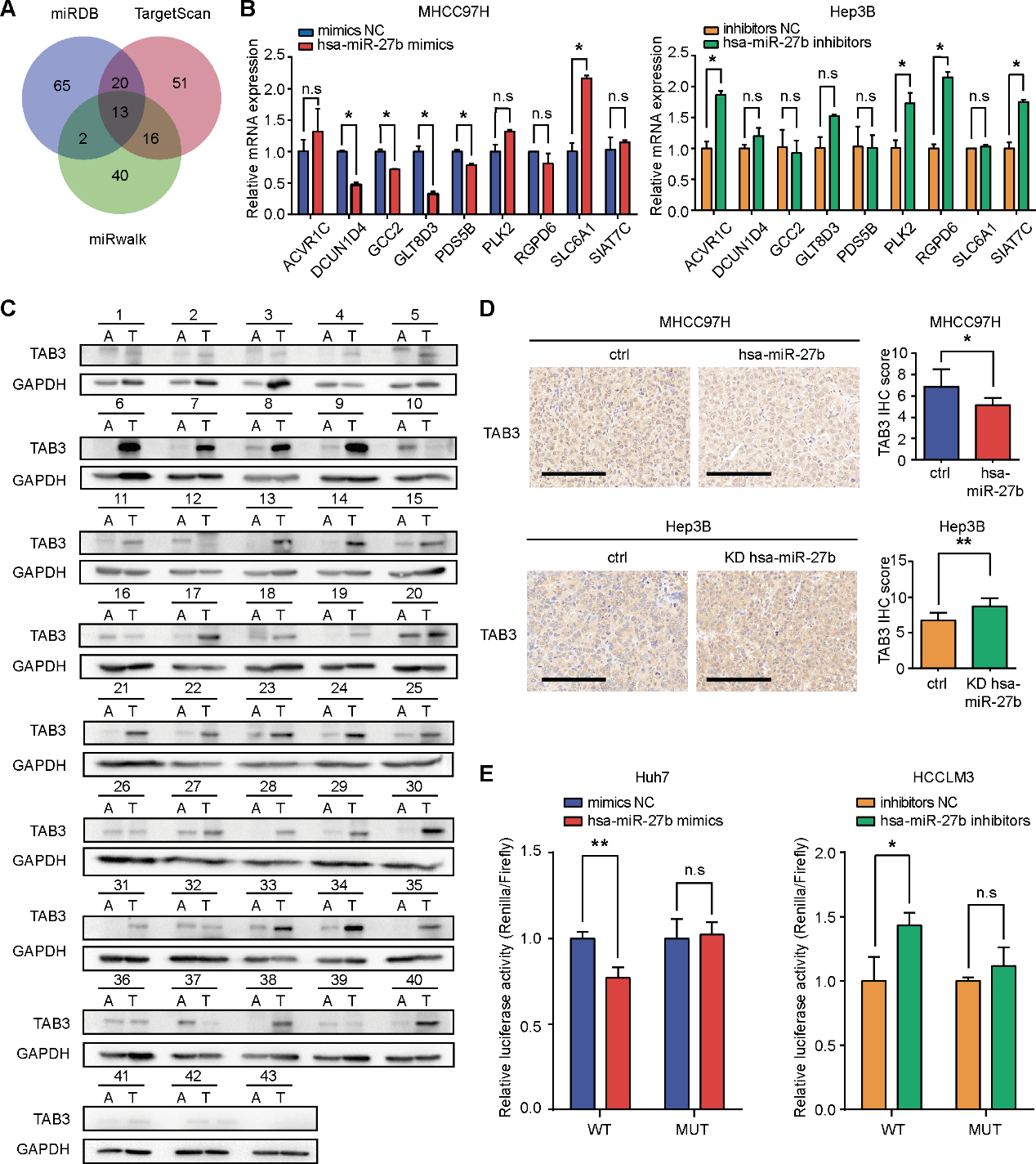
**

**Figure S3**

**
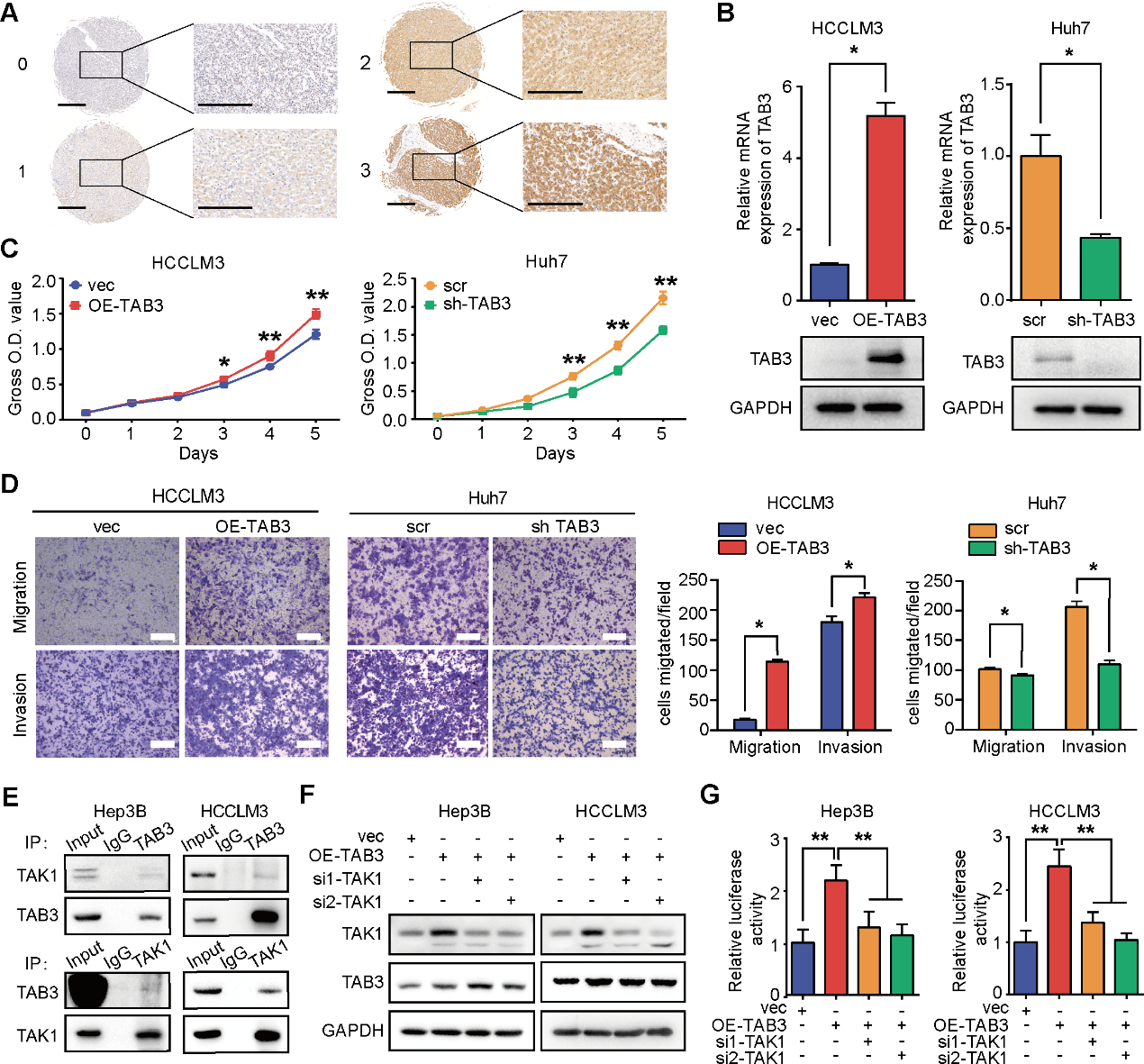
**

**Figure S4**

**
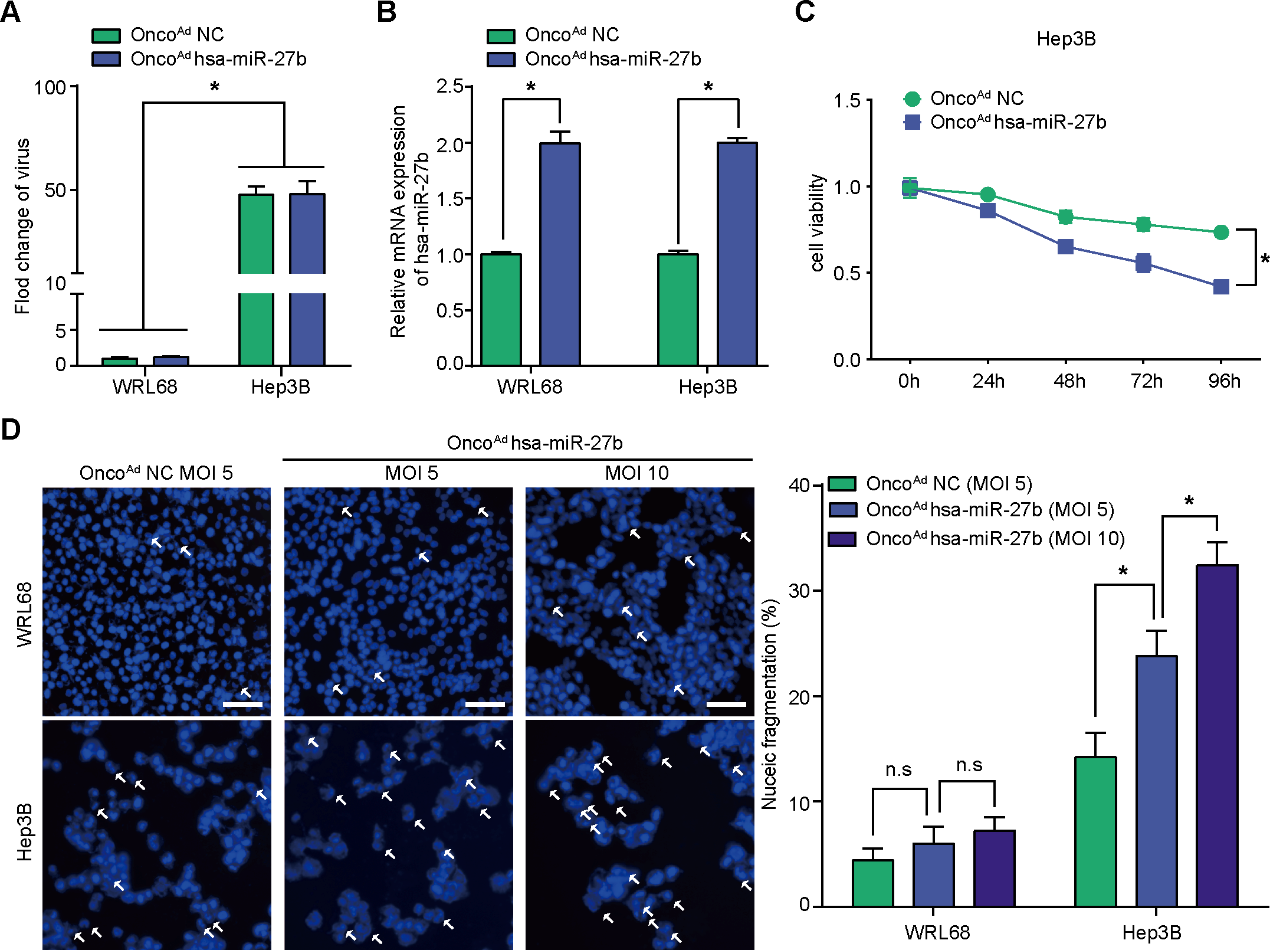
**

**Additional figure legends:**

**Figure S1**

(A) Representative images of ISH analysis for hsa-miR-27b expression scoring as 0, 1, 2 and 3 for staining intensity; Scale bar: overview images, 100 μm; magnified images, 200 μm.

(B-E) The indicated cells were transfected with mimics or inhibitors of hsa-miR-27b.

(B) CCK-8 assay for the indicated cells (n=5, Krustal-Wallis test).

(C) Representative images of EdU staining for the indicated cells and quantification of EdU positive cells; Scale bar: 100 μm (n=3, Mann-Whitney test).

(D) Representative images and quantification of cells migrated and invaded. Scale bar, 200μm (n=3, Mann-Whitney test).

(E) Representative images of phalloidin staining for F-actin (red) in the indicated cells. DAPI was used to show the nucleus (blue). Scale bar, 20μm.

(B-E) were repeated for three times. *p < 0.05, **p < 0.01. Data are shown as Mean ± SD. Abbreviations: NC, negative control.

**Figure S2**

(A) Venn diagram analyses of the target mRNA of hsa-miR-27b from the miRDB, miRWalk and Targetscan.

(B) qRT-PCR assay was performed to detect the mRNA level of the indicated genes in MHCC97H cells treated with hsa-miR-27b mimics, or in Hep3B cells treated with hsa-miR-27b inhibitors. Data were normalized to GAPDH and are shown as the fold change to their respective control cells (n=3, Mann-Whitney test).

(C) Western blot analysis of TAB3 expression in HCC and ANTs specimens. GAPDH as loading control.

(D) Representative IHC images and quantification of TAB3 scoring in subcutaneous xenografts. Scale bar, 200 μm (n=7, unpaired t-test).

(E) Dual luciferase reporter assay of cells co-transfected of Wild-type (WT) or mutated TAB3 3’-UTR luciferase reporter plasmids with hsa-miR-27b mimics or inhibitors in Huh7 and HCCLM3 cells (n=3, Mann-Whitney test).

(B and E) were repeated for three times. *p < 0.05, **p < 0.01. Data are shown as Mean ± SD. Abbreviations: NC, negative control; A, adjacent nontumorous tissues; T, tumor; ctrl, control; KD, knocking down; MUT, mutation.

**Figure S3**

(A) Representative IHC staining intensity scores of TAB3; Scale bar: overview images, 100 μm; magnified images, 200 μm.

(B) qRT-PCR (upper panel) and western blot (lower panel) analysis of TAB3 overexpression or knocking down efficiency in HCCLM3 or Huh7 cells. Data were normalized to GAPDH and are shown as the fold change to their respective control cells for qRT-PCR (n=3, Mann-Whitney test). GAPDH as loading control for western blot analysis.

(C) CCK-8 assay of the indicated cells (n=5, Krustal-Wallis test).

(D) Representative images of cell migration assay and invasion assay in the indicated HCC cells. Scale bar, 200 μm. Quantification of cell migrated and invaded (n=3, Mann-Whitney test).

(E) Co-IP assay for the binding between TAB3 and TAK1 in the TNF-α (10ng/ml) treated Hep3B and HCCLM3 cells.

(F) Western blot analysis of TAK1 and TAB3 protein level in indicated Hep3B and HCCLM3 cells. GAPDH as loading control.

(G) Dual luciferase reporter analysis for NF-кB transcription activity in the TNF-α (10ng/ml) treated cells. Data were normalized to their respective negative control cells. (n=3, One-way ANOVA test).

(B-G) were repeated for three times. *p < 0.05, **p < 0.01. Data are shown as Mean ± SD. Abbreviations: vec, vector; OE, overexpressing; scr, scramble; sh, small hairpin RNA. si, small interference RNA.

**Figure S4**

(A) Analysis of the replication ability of the oncolytic adenoviruses in normal hepatocytes (WRL68) and tumor cells (Hep3B) by qRT-PCR. The adenovirus E1A region was amplified to evaluate viral replication 48 hours after infection (MOI of 10) (n=3, Mann-Whitney test).

(B) The expression of hsa-miR-27b expression was measured by qRT-PCR analysis after being infected with oncolytic adenoviruses (MOI of 10). Data were normalized to U6 and are shown as the fold change to their respective negative control cells (n=3, Mann-Whitney test).

(C) Hep3B cells were treated with recombinant oncolytic adenoviruses Onco^Ad^ hsa-miR-27b and Onco^Ad^ NC at 10 MOIs, the cell viabilities are evaluated with CCK-8 assay every 24 hours after infection (n=5, Krustal-Wallis test).

(D) Nucleic fragmentation (indicated by white arrows) was observed in WRL68 and Hep3B cells after 2-day treatment of Onco^Ad^ NC and Onco^Ad^ hsa-miR-27b at the indicated MOI, detected by Hoechst staining; scale bar: 100 μm. Quantification of proportion of nucleic fragmentation (n=3, Mann-Whitney test).

All experiments were repeated for three times. *p < 0.05, **p < 0.01. Data are shown as Mean ± SD. Abbreviations: Onco^Ad^, Oncolytic adenovirus; NC, negative control; MOI, multiplicities of infection.

**Additional table:**

**Table S1. Antibodies used in this study.**

| **Antigens** | | **Manufacturers** | | **Application** |
| --- | --- | --- | --- | --- |
| ZO-1 | A0659, Abclonal, China | | 1:1000 for WB | |
| ZEB1 | A1500, Abclonal, China | | 1:1000 for WB | |
| N-cadherin | A19082, Abclonal, China | | 1:1000 for WB | |
| GAPDH | #5174, Cell Signaling Technology, USA | | 1:1000 for WB | |
| Ki67 | #2586, Cell Signaling Technology, USA | | 1:16000 for IHC | |
| TAB3 | ab85655, Abcam, USA | | 1:2000 for WB  1:100 for IP | |
| TAB3 | ab134806, Abcam, USA | | 1:400 for IHC | |
| AGO2 | ab32381, Abcam, USA | | 1:200 for RIP | |
| CCND1 | 60186-1-Ig, Proteintech, China | | 1:5000 for WB | |
| MMP9 | A0289, Abclonal, China | | 1:1000 for WB | |
| VEGFC | 22601-1-AP, Proteintech, China | | 1:1000 for WB | |
| c-myc | 67447-1-Ig, Proteintech, China | | 1:5000 for WB | |
| STAT3 | 10253-2-AP, Proteintech, China | | 1:1000 for WB | |
| TAK1 | 12330-2-AP, Proteintech, China | | 1:1000 for WB  1:100 for IP | |
| IKKβ | A2087, Abclonal, China | | 1:500 for WB | |
| p-IKKβ | Abs130638, Absin, China | | 1:1000 for WB | |
| IkB | A1187, Abclonal, China | | 1:1000 for WB | |
| p-IkB | AP0707, Abclonal, China | | 1:1000 for WB | |
| NF-κB (p65) | #6956, Cell Signaling Technology, USA | | 1:1000 for WB  1:400 for IHC  1:800 for IF | |
| Phospho-p65 (p-p65) | #3033, Cell Signaling Technology, USA | | 1:1000 for WB  1:400 for IHC  1:800 for IF | |
| Lamin A/C | A19524, Abclonal, China | | 1:1000 for WB | |

**Table S2. Primer sequences used for qRT-qPCR.**

| **Target** | **Primer sequence (5'-3')** |
| --- | --- |
| Hsa-miR-27b | Forward: ccgTTCACAGTGGCTAAGTTCTGC |
| U6 Small Nuclear RNA | Forward: AGCACATATACTAAAATTGGAACGAT |
| CCND1 | Forward: GCTGCGAAGTGGAAACCATC |
|  | Reverse: CCTCCTTCTGCACACATTTGAA |
| MMP9 | Forward: TGTACCGCTATGGTTACACTCG |
|  | Reverse: GGCAGGGACAGTTGCTTCT |
| VEGFC | Forward: CCCGCCTCTCCAAAAAGCTA |
|  | Reverse: CGGGTGTCAGGTAAAAGCCT |
| c-myc | Forward: GTCAAGAGGCGAACACACAAC |
|  | Reverse: TTGGACGGACAGGATGTATGC |
| STAT3 | Forward: CAGCAGCTTGACACACGGTA |
|  | Reverse: AAACACCAAAGTGGCATGTGA |
| TAB3 | Forward: AGCAGCCCACAGCTTGATATT |
|  | Reverse: ACTAGGAGAATGGATACCCAGGT |
| ACVR1C | Forward: TGAACAGGGCTCCTTATATGACT |
|  | Reverse: GTGTGCCAGACCACTAGCAA |
| DCUN1D4 | Forward: GCCGCCGCTGTCAATTTTC |
|  | Reverse: AGGTTCAGCTTATTAAGGGTGTG |
| GCC2 | Forward: AAAATGAAGCAAGAGGTTGAGGA |
|  | Reverse: GTATTTGGAACGTACTGCCATCA |
| GLT8D3 | Forward: TTCTTGGCACGTTTGGTGGA |
|  | Reverse: ACCACGCTCTACACAAGTCC |
| PDS5B | Forward: GATGTTCGCTTACTGGTAGCC |
|  | Reverse: TCTAGCCCCTTCAACTGTCTT |
| PLK2 | Forward: CTACGCCGCAAAAATTATTCCTC |
|  | Reverse: TCTTTGTCCTCGAAGTAGTGGT |
| RGPD6 | Forward: CTGCTTCAGTGGCTTGCAG |
|  | Reverse: ATTGACTTCTGTCGAGGCGA |
| SLC6A1 | Forward: AGGAGGTGCTTACCTGGTG |
|  | Reverse: AGCGGGTACTGGAAGGACTT |
| SIAT7C | Forward: TGCTGGTTGTGCGTCTTGTAA |
|  | Reverse: GCCTGTATGTGTAGGAGAATGGT |
| E1A | Forward: TTCTCCGGAGCCGCCTCACCTTT |
|  | Reverse: AGGCTCAGGTTCAGACACAG |
| GAPDH | Forward: GGAGTCCACTGGCGTCTTCA |
|  | Reverse: GTCATGAGTCCTTCCACGATACC |

**Table S3. Clinicopathologic characteristics of patients with HCC**

| Clinical characteristic | Number  n=71 | Percentage (%) | |
| --- | --- | --- | --- |
| Gender |  | |  |
| Male | 57 | 80.28 | |
| Female | 14 | 19.72 | |
| Age (years) |  |  |  |
| ≤50 | 42 | 59.15 | |
| >50 | 29 | 40.85 | |
| ALT (U/L) |  |  |  |
| ≤70 | 65 | 91.59 | |
| >70 | 6 | 8.41 | |
| GGT (U/L) |  |  | |
| ≤54 | 37 | 52.11 | |
| >54 | 34 | 47.89 | |
| AFP (ng/ml) |  |  | |
| ≤400 | 40 | 56.33 | |
| >400 | 31 | 43.67 | |
| Tumor size (cm) |  |  | |
| ≤5 | 39 | 54.93 | |
| >5 | 32 | 45.07 | |
| Tumor number |  |  | |
| Single | 60 | 84.51 | |
| Multiple | 11 | 15.49 | |
| BCLC |  |  | |
| 0+A | 53 | 74.65 | |
| B+C | 18 | 25.35 | |
| HBsAg |  |  | |
| Negative | 14 | 19.72 | |
| Positive | 57 | 80.28 | |
| Tumor encapsulation |  |  | |
| None | 25 | 35.21 | |
| Complete | 46 | 64.79 | |
| Cirrhosis |  |  | |
| No | 7 | 9.86 | |
| Yes | 64 | 90.14 | |
| Satellite nodules |  |  | |
| No | 55 | 77.46 | |
| Yes | 16 | 22.54 | |
| Child-Pugh |  |  | |
| A  B | 63 | 88.73 | |
|  | 8 | 11.27 | |
| Microvascular invasion |  |  | |
| No | 60 | 84.51 | |
| Yes | 11 | 15.49 | |
| Macrovascular invasion |  |  | |
| No | 64 | 90.14 | |
| Yes | 7 | 9.86 | |
| Ascites |  |  | |
| No | 57 | 80.28 | |
| Yes | 14 | 19.72 | |

**Table S4. Correlation between relative hsa-miR-27b expression and clinical characteristic in HCC patients (n=71).**

| Clinical characteristic | Hsa-miR-27b expression  Low (n=37) High (n=34) | | P value |
| --- | --- | --- | --- |
| Gender |  | | |
| Male | 32 | 25 | 0.170 |
| Female | 5 | 9 |  |
| Age (years) |  | | |
| ≤50 | 21 | 21 | 0.668 |
| >50 | 16 | 13 |  |
| ALT (U/L) |  | | |
| ≤70 | 35 | 30 | 0.919* |
| >70 | 2 | 4 |  |
| GGT (U/L) |  |  |  |
| ≤54 | 18 | 19 | 0.542 |
| >54 | 19 | 15 |  |
| AFP (ng/ml) |  |  |  |
| ≤20 | 11 | 18 | **0.047** |
| >20 | 26 | 16 |  |
| AFP (ng/ml) |  |  |  |
| ≤400 | 14 | 26 | **0.002** |
| >400 | 23 | 8 |  |
| Tumor size (cm) |  |  |  |
| ≤5 | 19 | 20 | 0.524 |
| >5 | 18 | 14 |  |
| Tumor number |  |  |  |
| Single | 31 | 29 | 0.861 |
| Multiple | 6 | 5 |  |
| BCLC |  |  |  |
| 0+A | 26 | 27 | 0.376 |
| B+C | 11 | 7 |  |
| HBsAg |  |  |  |
| Negative | 5 | 9 | 0.170 |
| Positive | 32 | 25 |  |
| Cirrhosis |  |  |  |
| No | 2 | 5 | 0.181* |
| Yes | 35 | 29 |  |
| Satellite nodules |  |  |  |
| No | 27 | 28 | 0.345 |
| Yes | 10 | 6 |  |
| Child-Pugh |  |  |  |
| A  B | 30 | 33 | **0.036*** |
|  | 7 | 1 |  |
| Microvascular invasion |  |  |  |
| No | 25 | 25 | 0.220 |
| Yes | 12 | 9 |  |
| Macrovascular invasion |  |  |  |
| No | 32 | 32 | 0.251* |
| Yes | 5 | 2 |  |
| Ascites |  |  |  |
| No | 29 | 28 | 0.674 |
| Yes | 8 | 6 |  |

Bold number indicates P value<0.05; * indicates Fisher-exact test.

**Table S5. Univariate and multivariate analysis of factors associated with overall survival of 71 HCC patients**

|  | **Survival** | | | | | |
| --- | --- | --- | --- | --- | --- | --- |
|  | **Univariate analysis** | | | **Multivariate analysis** | | |
|  | **HR** | **95%CI** | **P value** | **HR** | **95%CI** | **P value** |
| **Gender (Male vs Female)** | 1.888 | 0.560 - 6.357 | 0.305 |  |  |  |
| **Age (>50 vs ≤50)** | 0.855 | 0.377 - 1.939 | 0.708 |  |  |  |
| **Serum AFP (>400 vs ≤400ug/L)** | 2.115 | 0.914 - 4.891 | 0.080 |  |  |  |
| **ALT (>75 vs ≤75 U/L)**  **GGT (>54 vs ≤54 U/L)**  **HBV (Positive vs Negative)**  **Cirrhosis (Yes vs No)** | 1.015  1.970  1.569  1.131 | 0.137 - 7.534  0.851 - 4.557  0.466 - 5.283  0.265 - 4.825 | 0.989  0.113  0.467  0.868 |  |  |  |
| **Tumor size (>5 vs ≤5 cm)**  **Tumor number (Multiple vs Single)** | 2.249  1.872 | 0.973 - 5.199  0.694 - 5.050 | 0.058  0.216 |  |  |  |
| **Tumor encapsulation (Complete vs None)** | **0.220** | **0.093 - 0.520** | **0.001** | 0.434 | 0.156 – 1.211 | 0.434 |
| **Ascites (Yes vs No)** | **2.431** | **1.028 - 5.747** | **0.043** | **2.144** | **1.118 - 11.999** | **0.032** |
| **Macrovascular invasion (Yes vs No)**  **Microvascular invasion (Yes vs No)**  **Satellite nodules (Yes vs No)**  **Child-Pugh (B vs A)** | **4.593**  **2.749**  **2.840**  1.778 | **1.683 - 12.535**  **1.209 - 6.253**  **1.224 - 6.590**  0.604 - 5.231 | **0.003**  **0.016**  **0.015**  0.296 | **6.573**  0.922  **2.975** | **1.098 - 39.352**  0.293 - 2.897  **1.046 - 8.465** | **0.039**  0.889  **0.041** |
| **BCLC stage (B+C vs 0+A)**  **Hsa-miR-27b expression (High vs Low)** | **2.859**  **0.101** | **1.249 - 6.543**  **0.014 - 0.749** | **0.013**  **0.025** | 0.848  **0.121** | 0.257 - 2.796  **0.016 - 0.926** | 0.787  **0.042** |
